# Bone retouchers and technological continuity in the Middle Stone Age of North Africa

**DOI:** 10.1371/journal.pone.0230642

**Published:** 2020-03-30

**Authors:** Elaine Turner, Louise Humphrey, Abdeljalil Bouzouggar, Nick Barton

**Affiliations:** 1 Monrepos Archaeological Research Centre and Museum for Human Behavioural Evolution, RGZM, Neuwied, Germany; 2 Centre for Human Evolution Research, The Natural History Museum, London, United Kingdom; 3 Institut National des Sciences de l’Archéologie et du Patrimoine, Rabat-Instituts, Rabat, Morocco; 4 Department of Human Evolution, Max Planck Institute for Evolutionary Anthropology, Leipzig, Germany; 5 Institute of Archaeology, University of Oxford, Oxford, United Kingdom; Max Planck Institute for the Science of Human History, GERMANY

## Abstract

Evidence for specialised bone tools has recently been reported for the Middle Stone Age of North Africa [one], which complements similar finds of slightly younger age in South Africa [two, three]. However, until now scant reference has been made to lesser known tools also made of bone (‘bone retouchers’) that were employed specifically as intermediaries for working or refining stone artefacts, that are sometimes present in these assemblages. In this paper we describe 20 bone retouchers from the cave of Grotte des Pigeons at Taforalt in north-east Morocco. This is the largest stratified assemblage of bone retouchers from a North African MSA site, and the biggest single collection so far from the African Continent. A total of 18 bone retouchers was recovered in securely dated archaeological levels spanning a period from ~ 84.5 ka to 24 ka cal BP. A further two bone retouchers were found in a layer at the base of the deposits in association with Aterian artefacts dating to around 85,000 BP and so far represent the earliest evidence of this type of tool at Taforalt. In this paper we present a first, detailed description of the finds and trace the stages of their production, use and discard (*chaîne opératoire*). At the same time, we assess if there were diachronic changes in their form and function and, finally, explore their presence in relation to stone tools from the same occupation layers of the cave.

## Introduction

Palaeolithic research has shown that the utilisation of tools made of animal tissues, such as bone, but also teeth, antler and ivory, has a long history, with their earliest known appearance in East Africa between 2.1–1.15 Ma BP (S1). Over this long time span, various types of these objects—commonly referred to as “bone tools”—have been recognised, indicating they were utilised for different functions [1]. The spectrum is broad, ranging from formal bone tools which were deliberately shaped or worked to produce a tool with a particular function in mind–for example projectile point; awl; pin, knives, to pieces of bone that seem to have been selected because their natural shape or size or both was considered suitable for use without any further, or only minimal, modification. Unmodified bones used as hammers [2,3] as anvils [4] and fragments of animal bone used as retouchers fall mainly into the latter category.

Bone retouchers are defined in this paper as shaft (diaphyseal) fragments of long bones bearing distinctive areas of impacts and abrasions on their surfaces. They were used as intermediaries to shape and/or refine stone tools by percussion or pressure and replaced stone hammerstones in the lithic *chaîne opératoire* [1]. According to Mozota [5:28] bone retouchers form “a conceptual bridge between the procurement of faunal resources and the management of mineral resources …, providing vital information for understanding how faunal and lithic management are integrated into the overall subsistence strategy “.

In western Europe, similar bone retouchers were already known from the 19^th^ century from Middle Palaeolithic contexts at Trou Magrite (Belgium) [6] and at La Quina (France) [7,8]. Since then bone retouchers have been identified in many assemblages dating from the Lower, Middle, and Upper Palaeolithic as well as from the Mesolithic and Neolithic. Geographically, the distribution of these tools extends across Europe [9,10] to the Levant [11] and the Altai Mountains [12], and recently even as far east as Lingjing in China [13].

**Fig 1:**
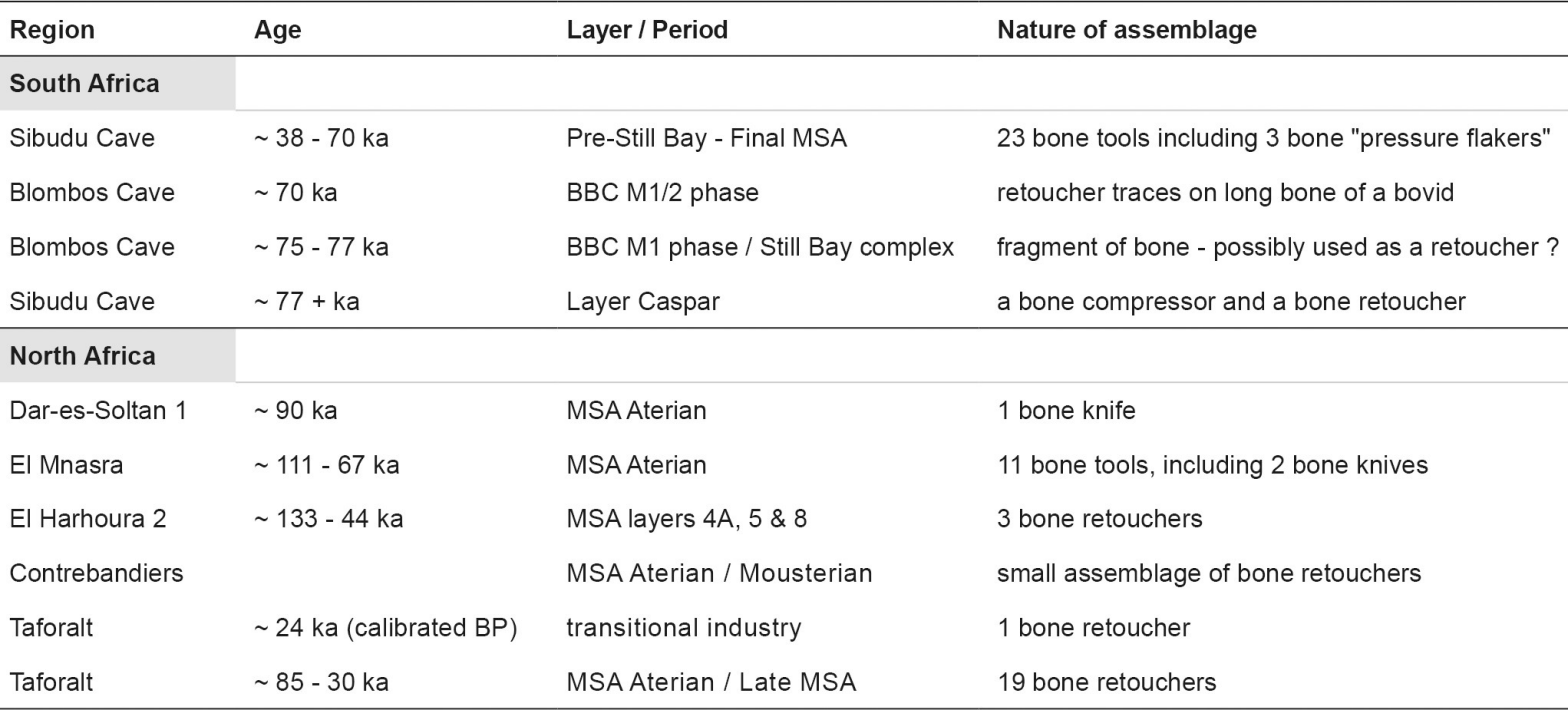
Summary of assemblages of bone tools from sites in South and North Africa.

The presence of bone retouchers in Africa is less well established (Fig 1). One is known from Blombos Cave, from the BBC M1/2 phase dating to around 70 ka and is a bovid long bone shaft that bears distinctive traces of use as a retoucher on the outer surface towards the apex and base [14: Appendices A1 and A2, SAM-AA No. 8950, “percussor”]; for a fuller description see S1. Less certain is another midshaft fragment from the underlying BBC M1 phase (Still Bay Complex), dating to around 75–77 ka, which has wear patterns consistent with multiple purposes [15] that may have included use as a retoucher. At Sibudu Cave, amongst 23 bone tools from layers spanning Pre-Still Bay to Final MSA (approximately ~ 70 ka BP– 38 ka BP [16,17] are three broken bone flakes with naturally pointed ends displaying traces of use and resharpening on the tips. Comparable modifications were produced on experimental pieces used as pressure flakers, suggesting similar activities produced these features on the archaeological finds [16]. A fragment of a bone compressor and a bone percussion tool were recovered from a single layer (layer Caspar) in a context older than 77 ka BP at Sibudu Cave [18: Fig 14]. These finds were in association with quartzitic bifacial tools. The percussive tool bears scores and scrapes on the surface of the bone comparable to the traces identified in this paper as retoucher damage and has a quartzite flake fragment embedded in one of the scores [18]. For North Africa the record is equally sparse. Despite the recovery of shaped bone tools (knives) from MSA Aterian layers at Dar es-Soltan 1 and El Mnasra [19,20], no examples of bone retouchers are known at these sites. Three bone retouchers have been reported at El Harhoura 2 from MSA layers (4A, 5 and 8) [21: Fig 147, Table 123]. Unfortunately, the dating of the MSA at El Harhoura 2 is problematic (S1). A small number of unpublished bone retouchers has also been identified at Contrebandiers from MSA contexts (pers. comm. 2018, Emily Hallet-Desguez).

In this paper we demonstrate a more extensive archaeological record of bone retouchers in the MSA of North Africa by presenting finds from the cave of Grotte des Pigeons at Taforalt in north-east Morocco. A total of 18 bone retouchers was recovered in securely dated archaeological levels at Taforalt spanning a period from ~ 84.5 ka to 24 ka cal BP. This sequence of layers covers MSA Aterian to late MSA levels and includes one retoucher from a potentially post-MSA, transitional context. A further two bone retouchers found in a layer at the base of the deposits in association with Aterian artefacts date to around 85,000 BP and represent the earliest evidence of this type of tool at Taforalt.

### Archaeological and stratigraphical context

**Fig 2:**
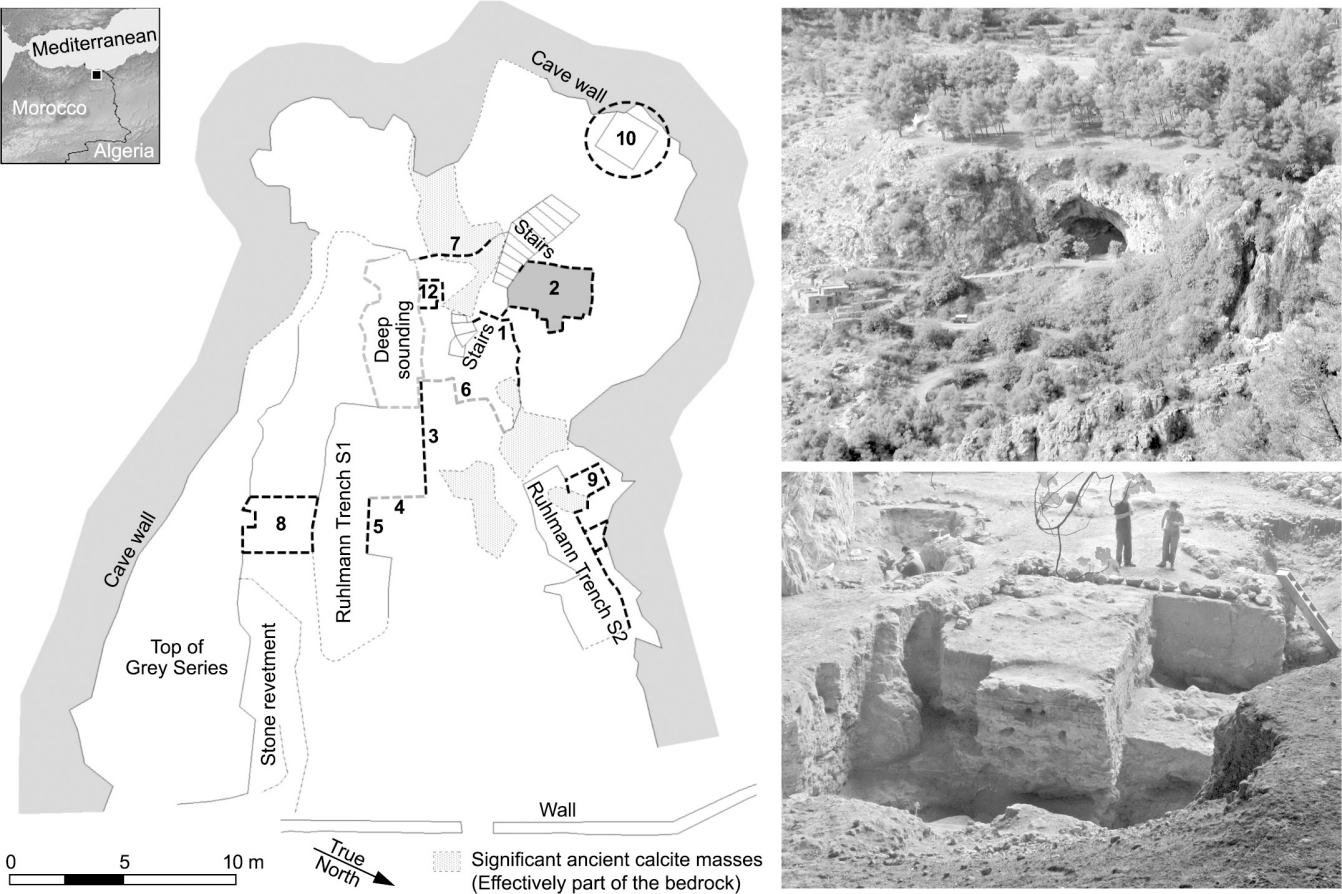
Location of the grotte des pigeons at Taforalt, in north-east morocco. Left: Plan of the cave interior, showing the different areas excavated during earlier field seasons directed by Roche (deep sounding) and Ruhlmann and sectors and sections investigated during our investigations (2–10). Sector 2 indicated by shaded area. Above right: View of the entrance to the cave. Below right: View of interior of cave with soundings.

Grotte des Pigeons (34° 48’ 50”N, 2° 24’ 14”W) also known as Taforalt Cave is located in north-eastern Morocco, some 40km from the Mediterranean coast (Fig 2). The cave lies at an elevation of 720m above sea level; it has a large north-east facing entrance and a floor area which currently measures just over 400m^2^. Previous excavations by Ruhlmann (1944–1947) and then Roche (1950–1955 and 1969–1977) confirmed the presence of a 4m sequence of Iberomaurusian Later Stone Age (LSA) deposits sealing >6m of Aterian and other Middle Stone Age (MSA) layers [22,23,24]. The cave also contains the largest LSA cemetery in North Africa [25].

A program of new excavations began in 2003 involving a collaboration between the Moroccan Institut National des Sciences de l'Archéologie et du Patrimoine and the University of Oxford (UK) [26]. All necessary permits were obtained for the described study, which complied with all relevant regulations.

The aim of this work was to reinvestigate the nature and dating of the archaeological deposits of the cave, to identify any major hiatuses in occupation as well as long-term trends in environmental and climatic change. The excavation sectors we investigated in the cave were deliberately opened adjacent to earlier trenches and close to the central type-section, thus enabling correlations with the original bed terminology, proposed by Raynal [27]. One of the key archaeological areas opened was designated Sector 2, where evidence of mainly MSA occupations in the cave was revealed (Fig 2).

**Fig 3:**
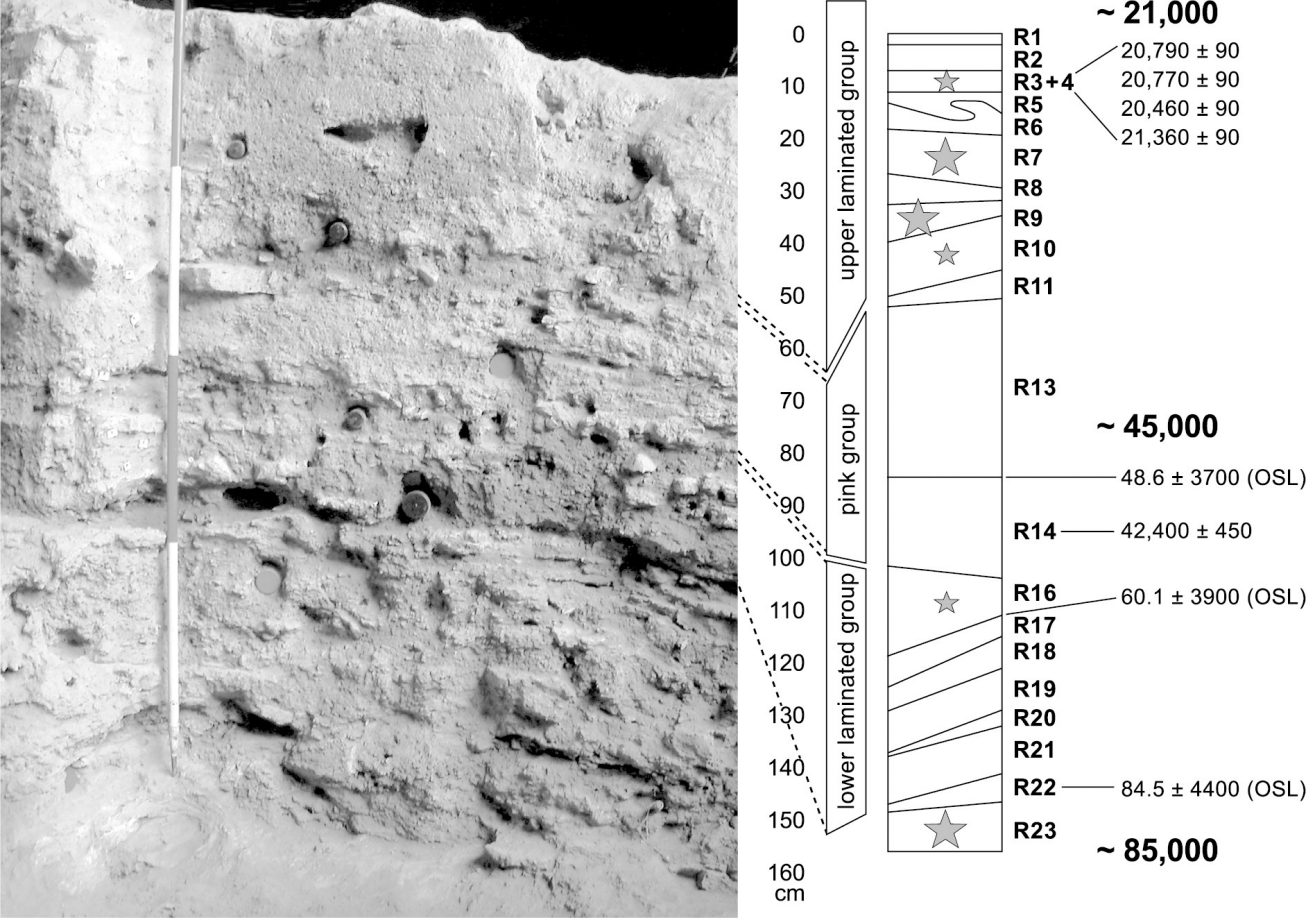
Sector 2 showing the three main units (Lower Laminated Group, Pink Group and Upper Laminated Group). R1 – R23 after Raynal [27]. Star symbols represent the location of the retouchers in the different layers: small stars represent one retoucher; larger stars represent several finds.

The MSA horizons in Sector 2 are characterised by two sets of finely laminated sediments (Lower and Upper Laminated Groups) with more homogenous sediments (Pink Group) stratified between the two [28]. Accelerator mass spectrometry (AMS), radiocarbon and OSL techniques were used to date the deposits in this sector [29] and the results of these determinations are shown in Fig 3.

**Fig 4:**
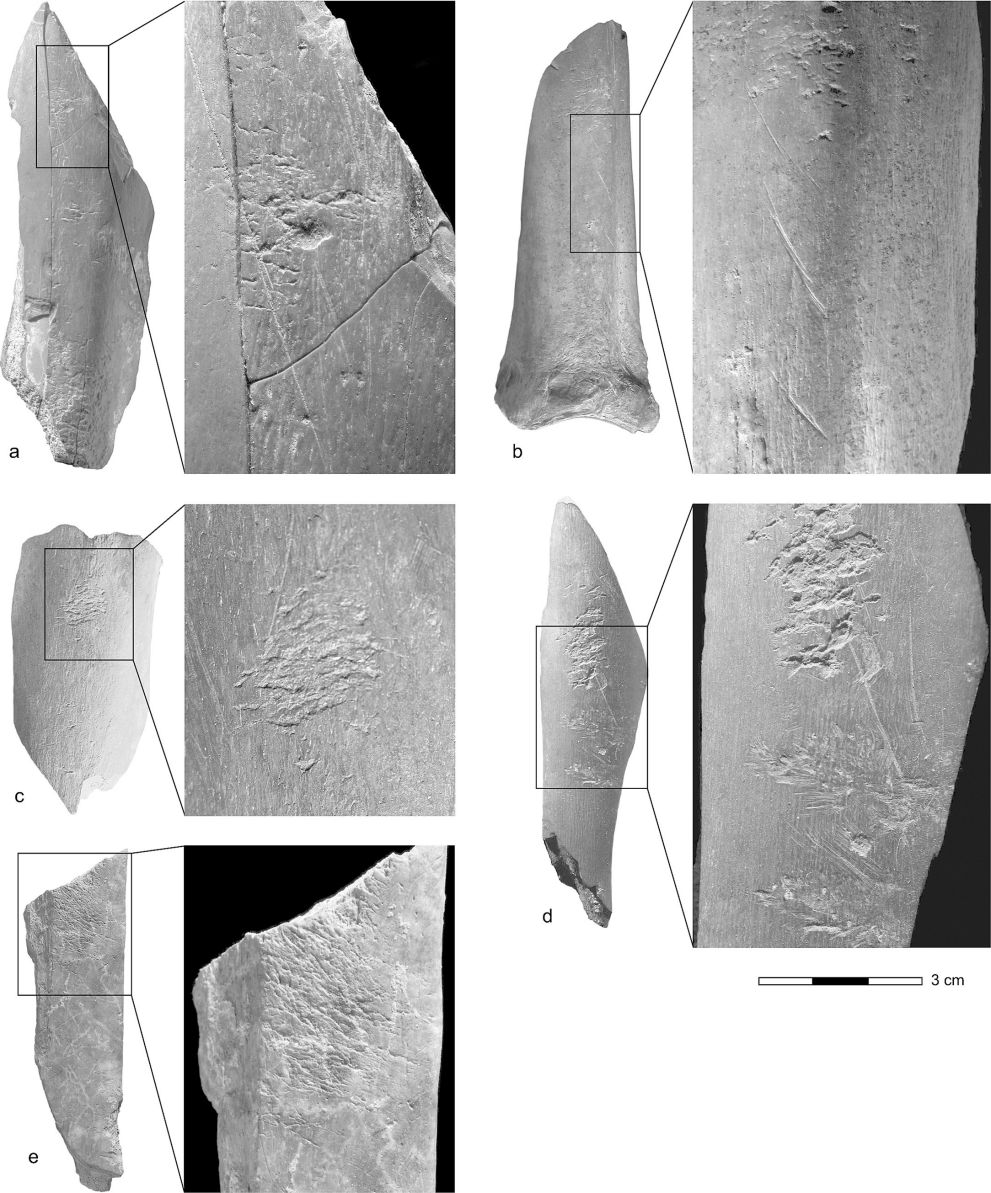
Bone retouchers from Taforalt. a: Transitional, layer R3-4 (TAF04-757). b: Late MSA, layer R7 (TAF05-2625). c: Late MSA, layer R9 (TAF04-1227). d: Late MSA, layer R9 (TAF05-2491). e: MSA Aterian, layer 23 (TAF10-10172).

Archaeological finds and hearth spreads are recorded in many of the units (S2). Tools of the Aterian facies of the MSA are present in layers R16–22 of the Lower Laminated Group and in layer R23. A late MSA without tanged points industry was recovered in layers R10—R5 of the Upper Laminated Group and an as yet unassigned industry with simple core adzes [30] in layers R3–4, in the uppermost part of the Upper Laminated Group surviving in Sector 2. Bone retouchers have been recovered from layers R3-4, R7, R9, R10, R16 and R23c and e (Figs 3 and 4).

## Materials and methods

**Fig 5:**
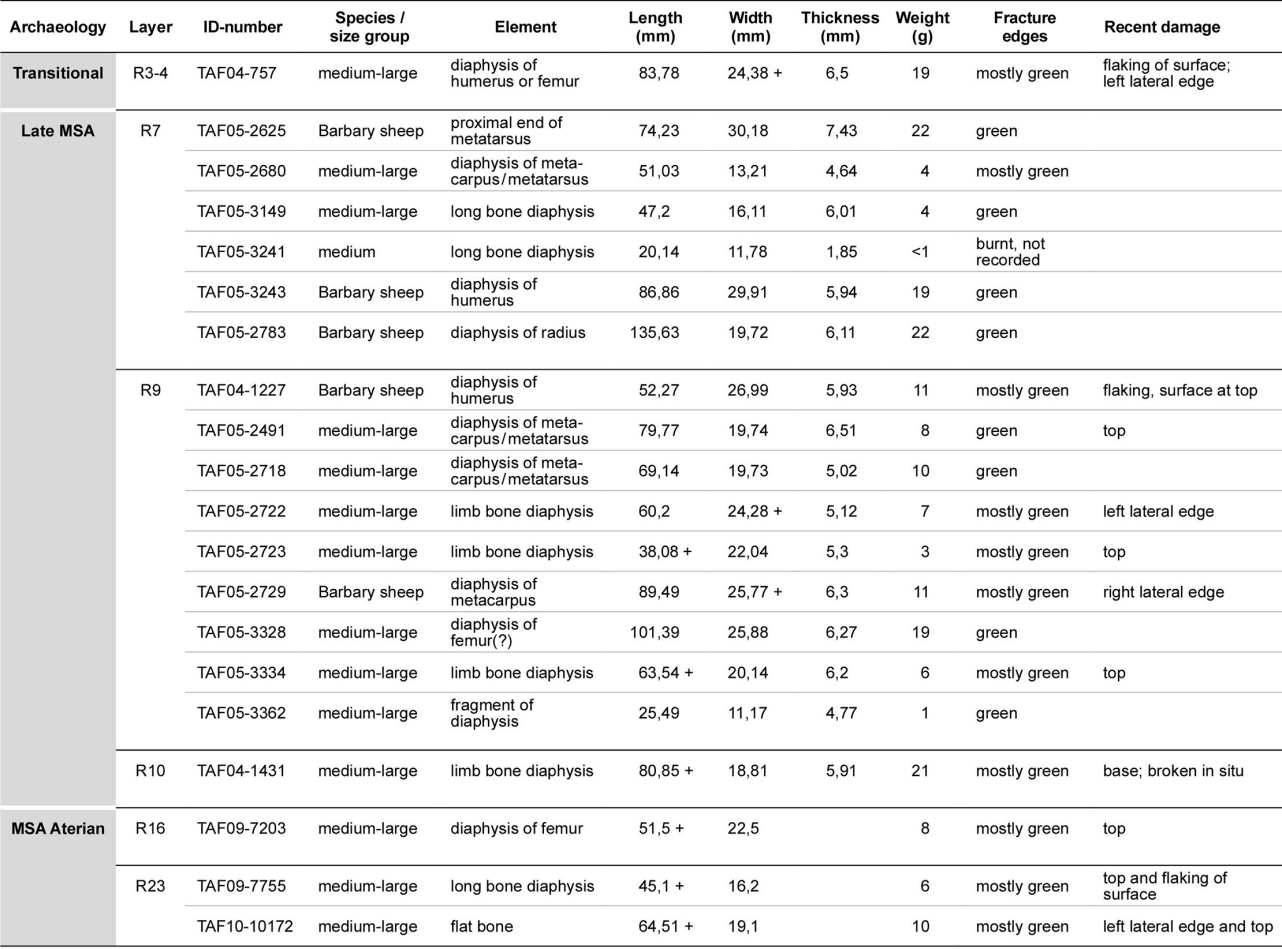
Description of the retouchers from Taforalt, showing taxonomy, element, dimensions, details of fractures and recent damage. mm: millimetres; g: grams.

**Fig 6:**
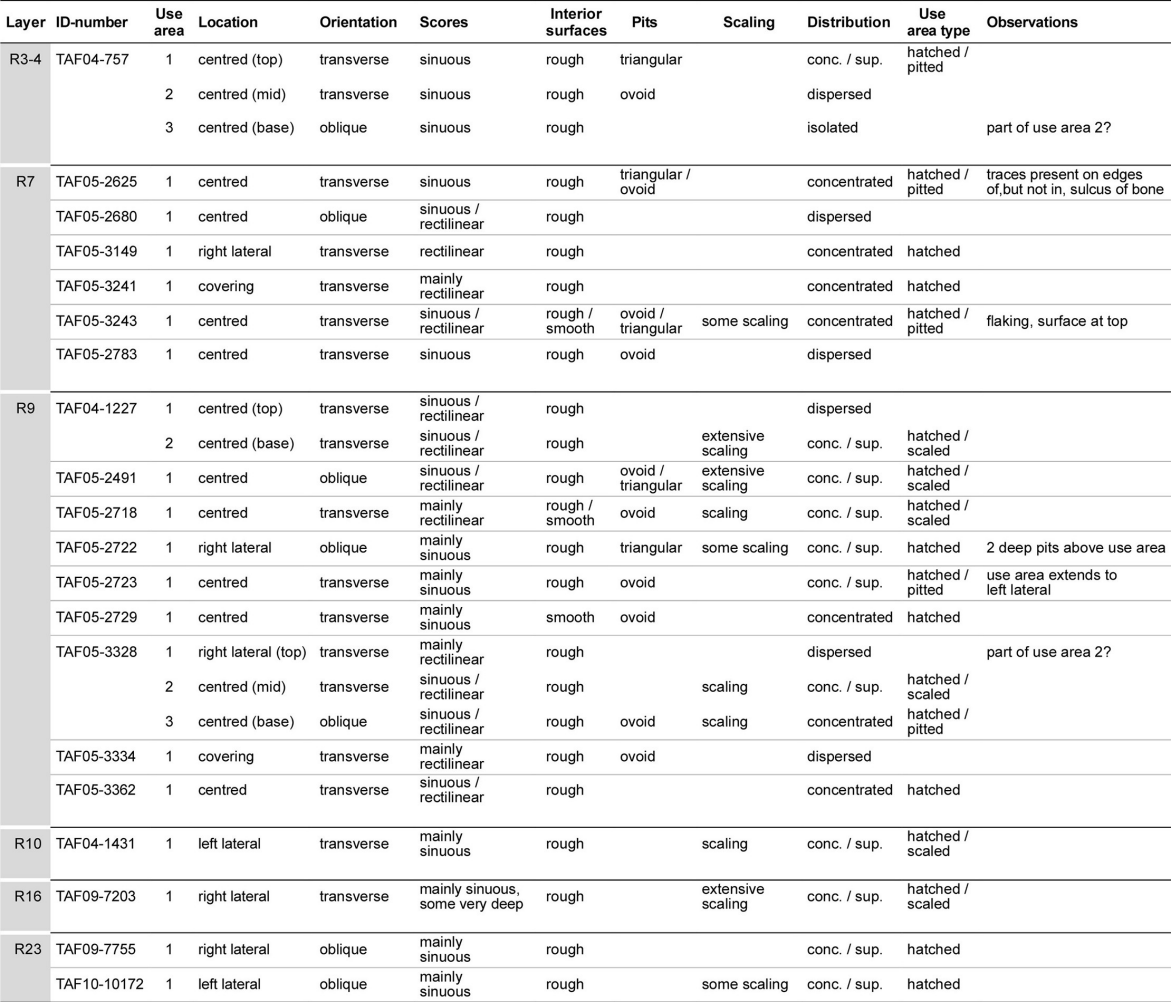
Location and description of use-areas on the retouchers from Taforalt. conc. / sup.: concentrated / superimposed.

The taxonomical and zooarchaeological analysis of the faunal remains (S3) from the 2005–2015 excavations was carried out by one of the authors (ET) and it was during these studies that the retouchers were recognised. The bone retouchers were recorded in the main faunal data-base, but were also entered into a second, smaller data-base which included the basic data (Fig 5) and additional criteria specific to the use-areas on the retouchers (Fig 6).

**Fig 7:**
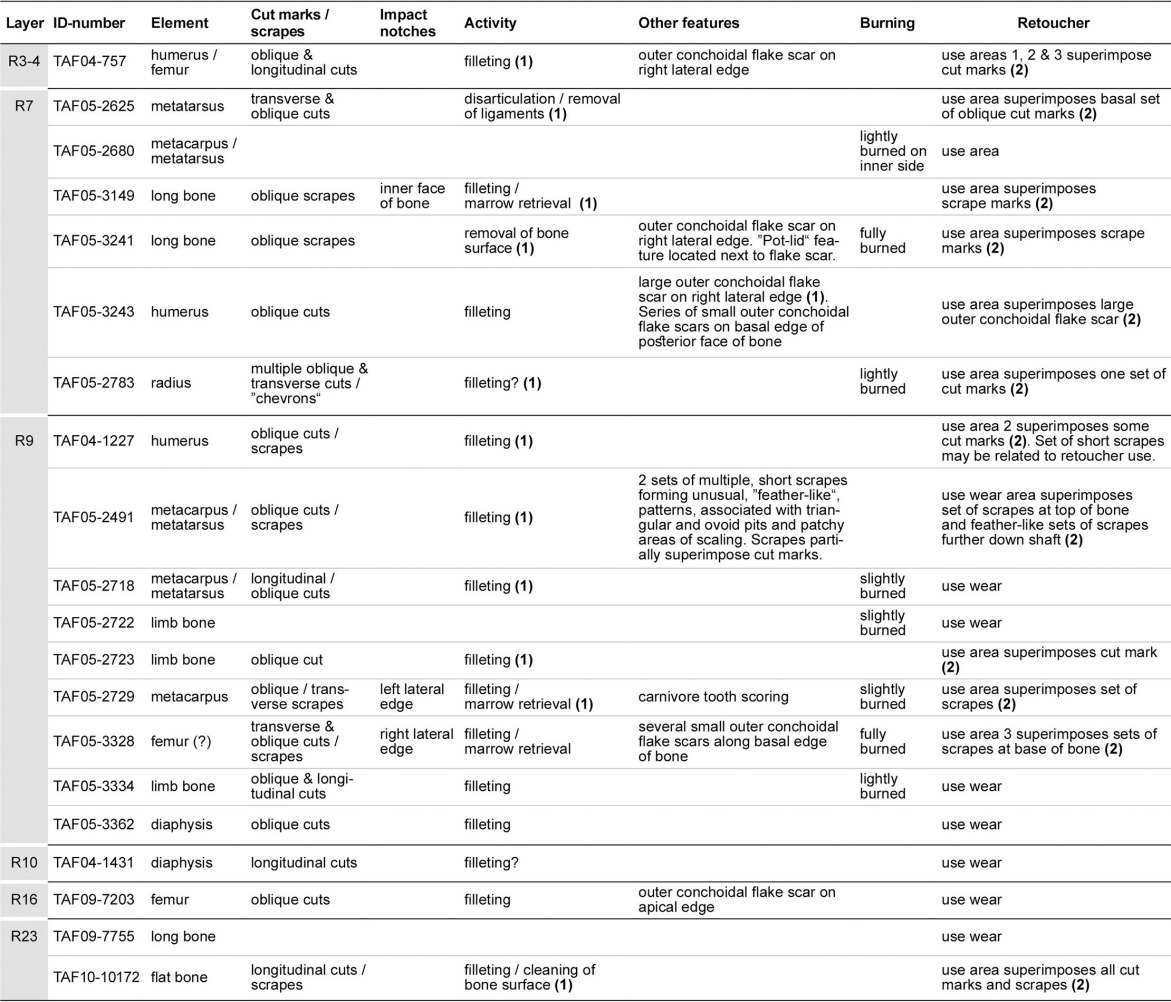
Compilation and interpretation of all traces observed on the bone retouchers from Taforalt. Bold numbers in parentheses indicate the running-order of stages of use of the bone blanks.

The description of the Taforalt finds followed both terminology and conventions proposed by Taute [31] and, in particular, those recently suggested by Mallye and others [32] with some minor adjustments to accommodate our material. An illustration of the orientation and conventions applied to the Taforalt finds is shown in S1 Fig. Establishing the history of use of the Taforalt retouchers was an important part of the analysis of these finds and sequences of utilisation were identified by observing superimposed signatures of modification on the finds (Fig 7).

Ten of the bone retouchers were 3-D scanned at i3mainz (University of Applied Sciences, Mainz) using a structured light scanner ATOS II (SO) from the firm GOM. Details of the modifications on the outer surfaces of these retouchers are illustrated on diagrams produced using the “snapshot” function of the programme MeshLab. Photographs of the retouchers were taken using a Nikon D2 x and a Canon Eos 30 D, all fitted with 60 mm Macro lens. The bone tools described in this paper are currently housed at the Laboratory of the National Institute of Archaeological Science and Heritage, Rabat (Morocco) and accessible to the scientific team members and to any authorized researcher.

## Results

A total of 20 bones bearing traces consistent with their use for retouching and re-sharpening lithic tools such as scrapers was recovered in Sector 2 at Taforalt. Three derive from the MSA Aterian deposits (layers R23 and R16), but the bulk of the retouchers (n = 16) are located in layers R10, R9 and R7, attributed to the late MSA (Fig 5). A single retoucher was recovered from layer R3 –R4, where an apparently transitional industry has been recognised [30].

### Selection of the blanks

Since seventeen of the bone retouchers bore traces of cut marks and impact notches deriving from marrow retrieval, it appears that bones selected for this purpose at Taforalt had been mainly sourced from the carcasses of animals that had been butchered.

Fragments of the diaphyses of long bones were primarily selected, comprising elements of the fore and rear limbs (humerus; radius; femur) and fore and rear feet (metatarsus; possibly metacarpus) (Fig 5). One retoucher from the MSA Aterian (TAF10-10172) (Fig 4E) is possibly a fragment of a flat bone. Five of the bone retouchers could be identified as bones of Barbary sheep and the bulk of the remaining finds are from the medium-large animal size group, to which Barbary sheep belongs. Only one retoucher in the late MSA assemblage (Layer R7, TAF05-3241) was tentatively identified to the medium animal size category.

Recent damage was observed on eleven of the retouchers and assessed for potential loss of bone with consequent reduction in the dimensions of the finds. Four pieces (TAF05-2491; TAF05-3334; TAF05-2723; TAF09-7203) did show loss of bone at the top of the find and might have originally been longer. However, in general, the damage on the finds was limited to thin splinters of bone lost along the top, basal and lateral edge(s), so the dimensions of the retouchers given in Fig 5 can be considered as close to their original sizes. Flaking of the bone surface did not seem to have influenced the dimensions of the finds at all.

The bone retouchers from Taforalt are not large (Fig 5), but show a wide range in size, particularly in the length. They range in length from around 20mm (late MSA, layer R7; TAF05-3241) to 135mm (late MSA, layer R7; TAF05-2783) and in breadth from 11mm (late MSA, layer R9; TAF05-3362) to 30mm (late MSA, layer R7; TAF05-2625). The smallest finds (TAF05-3241; TAF05-3362) are probably fragments of larger bone retouchers. Cortical thickness ranged from approximately 2mm (late MSA, layer R7; TAF05-3241) to 7mm (late MSA, layer R7; TAF05-2625) and the finds are not heavy, weighing between 1–22 grams.

**Fig 8:**
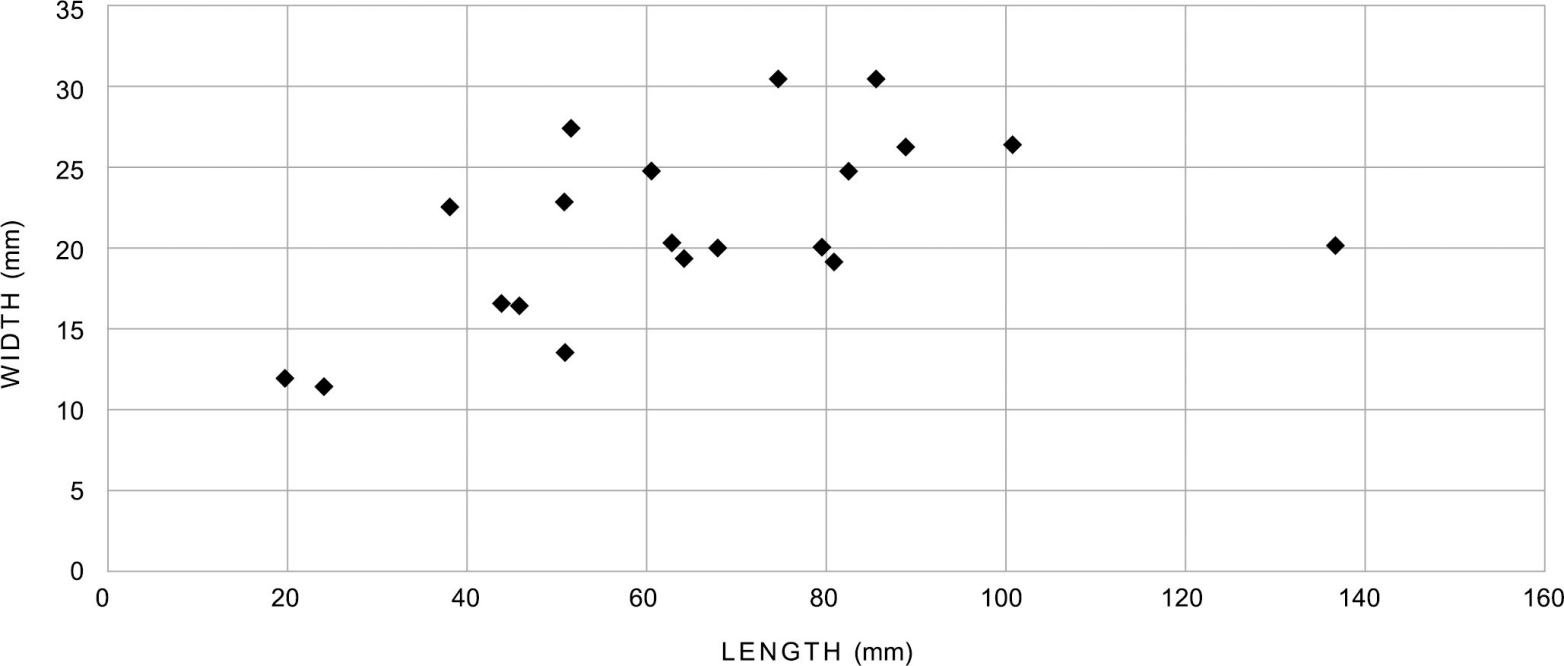
Length and breadth of the retouchers from Taforalt. (see Fig 5 for data).

**Fig 9:**
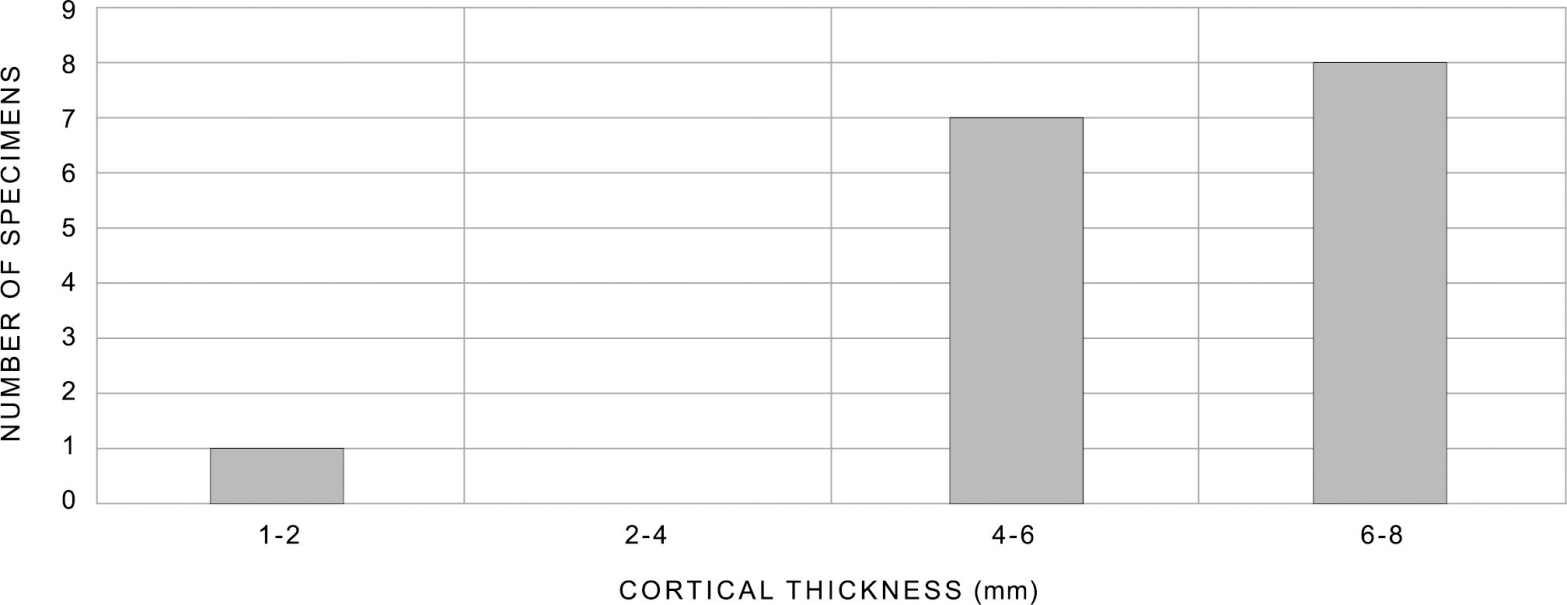
Cortical thickness of the retouchers from Taforalt. (see Fig 5 for data).

Plotting length and breadth of the bone retouchers (Fig 8) shows the majority of the finds clustering between approximately 40 – 100mm in length (average 70 mm) and 15 – 30mm in breadth. The three retouchers from the MSA Aterian and the single find from layers R3—R4 also fall within this size range. Although based on a very small number of finds (n = 4), this evidence might suggest that the dimensions of the retouchers did not change significantly through time. Only one bone retoucher (late MSA, layer R7; TAF05-2783), was longer than the other finds and fell outside the range of the main group. Cortical thickness is depicted in Fig 9, and shows, with one exception (late MSA, layer R7; TAF05-3241), that fragments 4 – 8mm thick were utilised.

It is interesting to note that bones of large animals, such as horse, large bovine or rhinoceros, and bones of animals in the large size group, all present in the Sector 2 faunal assemblages albeit in comparatively low counts, were not utilised as bone retouchers. Bone blanks at Taforalt appear to have been selected from what was at hand and abundant, namely the remains of the main game, the Barbary sheep and animals comparable in size to this species (S3 Table).

### Retoucher damage

The characteristics of the bone retouchers are listed in Fig 6, giving information on the individual use-areas on each blank and their distribution (number of areas; location; orientation), traces of retoucher damage (scores and their interior surfaces; pits; scaling) and, finally, types of use-areas.

### Use-areas and handedness

Seventeen retouchers from the MSA Aterian and late MSA display a single use-area and only three finds, one from the transitional layers R3—R4 (TAF04-757) (Fig 4A; S2 Fig) and two from the late MSA layer R9 (TAF04-1227) (Fig 4C; S5 Fig) (TAF05-3328) (S7 Fig), had been used more than once. Out of a total of 25 use-areas, 16 were centrally placed (Fig 6). The use-areas on two retouchers (TAF05-3334 and TAF05-3241) covered the bone from side to side.

Five use-areas were located on the right lateral and two on the left lateral edges of the bones. Interestingly, use-areas on the three retouchers from the MSA Aterian layers R16 and R23 are all positioned on the lateral edges of the bones rather than in the centre. The scores in seven use-areas are oriented oblique to the longitudinal axis of the bone fragment, but the bulk of the retouchers had been held or used in a way that consistently produced transverse scoring (n = 18 areas). A variable orientation was apparent on a find which had been used more than once (late MSA, layer R9; TAF05-3328, S7 Fig), where both transverse and oblique orientations of retoucher damage were recorded in individual use-areas on the same retoucher, suggesting either a change in the way the retoucher was held between phases of use or a change in techniques of utilisation of the same retoucher or a change in the user of the tool.

Right handedness is typically dominant in humans [33] and evidence of handedness has been seen in asymmetrically-retouched tools [34]. Semenov [35] suggested that certain features observed on bone retouchers from Middle and Upper Palaeolithic sites in Russia derived from their use by right-handed humans and a right-hand tendency was also observed on bone retouchers from the Middle Palaeolithic site of El Salt, Spain [36]. Mozota [5, 37](Fig 3) attributed a clear pattern of right lateralisation of use-areas on retouchers produced in his experimental programs to his right-handedness. This phenomenon has been observed during experiments undertaken by other researchers [38]. Malerba and Giacobini [39] also observed that the orientation of the scores in a use-area was associated with the use of a specific hand—a right-handed user produced scores with the bases oriented towards the right. Interestingly, no lateralisation, orientation of scoring and handedness, was observed by Mallye et al. [32] in their experimental work, while even Mozota [5:30] writes “..the criteria for identifying the lateralization of retouching tasks are not unified”.

Lateralisation was observed on only six of the retouchers from Taforalt with single use-areas: four of these are located on the right side and two on the left. Five of the Taforalt retouchers with single use-areas showed oblique scoring, but only three, (TAF05-2722; TAF09-7755; TAF10-10172, Fig 4E; S8 Fig), were observed in use-areas in lateral positions. The Taforalt retouchers can therefore neither confirm nor repudiate claims for lateralisation and handedness, since the bulk of the use-areas are located in central positions and the majority of the scores are transverse.

### Scores and pits and bone freshness

Both sinuous and rectilinear scores were identified, although sinuous scores clearly dominate and were recognised in 20 use-areas (Fig 6). In some cases sinuous and rectilinear scores were observed in a single use-area. The interior surfaces of the scores are mainly rough (n = 24 use-areas); smooth interior surfaces could only be observed in scores in three areas and in two of these areas they were also associated with scores with rough interior surfaces. Pits are present in twelve use-areas and scaling of the bone surface was observed in ten. Mallye et al. [32] conducted experiments using both fresh and defatted bones as retouchers. Although they found no relationship between the form of the scores and the pits and bone freshness, they did conclude that scaled areas were more commonly observed on defatted elements, even though the absence of scaling was not an indication that the bone was fresh [32: 1137]. This evidence suggests that ten of the Taforalt retouchers were utilised when the bones were not fresh. Mallye et al. [32] interpret the use of defatted bones as indicating repeated occupations of a cave by human group(s) over a relatively short period of time, where they found bones suitable as blanks for retouchers among the debris left behind from previous visits. The presence of ten retouchers on defatted bones could indicate a similar scenario at Taforalt, where raw material was collected from butchery debris produced during a previous visit or visits to the cave.

### Types of use-areas and their implications

Four types of distribution traces: isolated, dispersed, concentrated and concentrated and superimposed, were recognised by Mallye et al. [32] and all of these types of traces could be identified on the Taforalt finds (Fig 6). Eighteen of the use-areas on blanks from the MSA Aterian, late MSA and overlying levels are concentrated, or concentrated and superimposed. Six use-areas can be described as dispersed.

The continuing use of a retoucher results in the superimposition of traces of damage, producing three types of use-areas—hatched, pitted and scaled [32]. This was the case at Taforalt, where seven use-areas are hatched, five are hatched and pitted, and six are hatched and scaled. Mallye et al. [32] have shown correlations between types of use-areas and the type of raw material used in the production of the lithic artefacts. Thus, a high proportion of hatched areas was produced during the retouching of flint, whereas pitted areas are closely linked to working quartzite. At Taforalt the retouchers display mainly hatched areas, suggesting their use as tools for modifying the edges of lithic tools. This observation corresponds to the raw material spectrum of the artefact assemblages from Sector 2, which is dominated by flint and chert and very low counts of quartzite pieces.

In addition, in their experimental work, Mallye et al. [32: 1136] found a correlation between the distribution of the traces and the number of times a retoucher had been used. Use areas described as “dispersed” or “concentrated” were observed on retouchers applied less than 100 times to stone tools—on average 14 times. Use-areas with “concentrated and superimposed” traces were produced by the application of the retoucher more than 100 times—on average 123 times.

Thirteen of the use-areas on the Taforalt finds are dispersed or concentrated, suggesting these implements were not used intensively. At Taforalt, dispersed areas with only a few scores were located close to larger trace areas, indicating the former were possibly simply a by-product of retouching on a neighbouring portion of the bone. For example, the few traces in area 1 on the retoucher from the late MSA layer R9 (TAF05-3328), may have derived from minor peripheral damage produced while area 2 was in use. One of the use-areas on the blank from layers R3- R4 (TAF04-757 Fig 4A; S2 Fig) comprises an isolated score; and it is also possible that this mark is a peripheral scratch produced during the utilisation of area 2 on this find. A similar explanation can be postulated for two to three isolated scores, located close to the use-area on TAF05-2625 (Fig 4B; S3 Fig). The scores are located on the opposing sides of the sulcus (the pronounced channel on the dorsal face of the metatarsus, which marks the coalescence of the sutures of the metatarsals III and IV in the Cervidae and Bovidae). They were inflicted during the utilisation of area 1, and clearly show how the sharp edge of the lithic tool only came into contact with the protruding parts of this morphological feature.

Eleven of the use-areas on the retouchers from Taforalt are concentrated and superimposed, suggesting intensive use. There appears to be a strong correlation between higher numbers of retouchers and the presence of numerous, heavily worked side scrapers in the late MSA layers R7 and R9. However, contrary to the results of the experimental work described above, only six of these retouchers (all from layer 9) have use-areas displaying concentrated and superimposed traces associated with a more intensive use of the implement probably necessary to produce a heavily-worked artefact. In fact, with the exception of the bone retoucher from the MSA Aterian, layer R16 (TAF09-7203), where the scores are rather deep, none of the Taforalt finds bear a depression in the area of retoucher damage, typically found on bone tools which have been extremely heavily utilised during retouching [32,40].

### The production and use of the Taforalt retouchers (chaîne opératoire)

An interesting part of this analysis was retracing the steps involved in the production, use and discard of the retouchers from Taforalt. Reconstructing the *chaîne opératoire* of the bone blanks was possible due to the excellent preservation of the faunal remains from Taforalt, where signatures such as cut marks, impact notches, burning, and retoucher use wear and other features, are highly visible (Fig 7). Where relevant, the sequence of events which led to the deposition of the traces on the bone retouchers has been reconstructed by studying the superimposition of these features on the surfaces of the bones.

Only two bone retouchers (MSA Aterian, layer R23, TAF09-7755 and late MSA, layer R7; TAF05-2680) showed no traces of butchering activities. The remaining 18 blanks display cut marks, scrapes and notches deriving from carcass disarticulation (n = 1), filleting (n = 16), removal of ligaments (n = 1) and marrow retrieval (n = 3). On the whole, the numbers of cut marks, scrapes and notches did not reflect a particularly exhaustive butchering of these bones. Two tiny chips of stone from a lithic artefact were embedded in a cut mark produced during butchery on a fragment of a long bone from late MSA, layer R5 (TAF05-3020), but chips of stone were not observed in the scores or pits of the use-areas on the bone retouchers. Also absent on the Taforalt bone retouchers were multiple long, longitudinal and oblique scrapes indicating an intensive cleaning of the surface of the bone prior to its use as a retoucher. Similar scrapes have been commonly observed on bone retouchers from Middle Palaeolithic sites in Europe, such as Kůlna cave in Moravia [41].

Outer conchoidal flake scars were present on 5 bone retouchers. They occur as single flakes scars on three retouchers, or as a series of small, contiguous flake removals along one of the edges of the find (late MSA, layer R9; TAF05-3328 (S7 Fig). One retoucher from the late MSA assemblage in layer R7 bore both a single flake on the right lateral side and a set of smaller removals along part of its basal edge (TAF05-3243). This find was clearly utilised for retouching after the single flake had detached from the bone, since the surface of the negative of the flake also bears traces of retoucher damage (S4 Fig). Thus, it is likely that single flake removals were produced during “regular” butchering activities, such as opening the bone to obtain marrow. It is not clear whether the contiguous series of smaller flake removals on two of the bone retouchers were also produced during butchery. Their positions on the TAF05-3243 find (S4 Fig), are certainly not consistent with the idea that this piece of bone functioned as an end scraper, as postulated for the bone retoucher from Blombos Cave [15]. On the other hand, the position and form of contiguous flake removals along the basal edge of the TAF05-3328 bone retoucher (S7 Fig) can, in the absence of a microscopic study of the finds, only be tentatively suggested as resulting from other activities, possibly due to scraping or unknown taphonomic factors. In summary, areas of use on the retouchers overlaid cut marks on eleven of the finds; an additional bone had retoucher damage overlying an outer conchoidal flake removal (see above).

Eight retouchers showed varying degrees of burning from slight to fully burned. Burning probably took place after the blanks had been utilised, when the finds were discarded and deliberately dumped or accidentally incorporated into one of the fires lit in the cave during the accumulation of the deposits in Sector 2. A tiny, concave fracture is present on the surface of the small, fully burned, bone retoucher from the late MSA, layer R7 (TAF05-3241). The concave fracture is similar in appearance to “pot-lids”–thermal fractures occurring on stone artefacts [42]. Since the concave fracture has removed a portion of the use-area, it can be assumed that burning of this find, at least, definitely took place after the bone had been used for retouching.

Tooth scoring, produced in this case by a carnivore, was present on only one of the blanks (late MSA, layer R9; TAF05-2729). Since the tooth scores are isolated marks and bear no relationship to the anthropic signatures, it was not possible to establish which agent—hominins or carnivores—had accessed the bone first. However, considering the very low numbers of bones with carnivore (and rodent) gnawing traces from Sector 2 (a total of 3 finds), predominantly first access to the animal bones by hominins appears to be the more likely scenario.

## Discussion and conclusions

The evidence shows that the blanks at Taforalt were chosen from bones produced during the butchering of mainly Barbary sheep carcasses. Cut marks, scrapes, notches and outer conchoidal flakes, already present on the bulk of the bone fragments selected as retouchers, were overlain by damage produced during the retouching of chert artefacts. There was no evidence of any intensive cleaning of the bone surface prior to use, but some of the bones seem to have been selected when they were in a defatted state. Some of the blanks were used twice or maybe three times, but on the whole only once. There was no evidence of an extremely heavy utilisation of the retouchers, despite the presence of heavily retouched lithic artefacts in the late MSA layers. The bulk of the traces show that retouchers which were used only a few times and those used more often occur in more or less equal quantities. One retoucher may have also served as a scraper. Some of the retouchers broke either during use or subsequently and both intact retouchers and fragments of retouchers were discarded, with some becoming incorporated into fires lit in the cave. One find was slightly gnawed by a carnivore prior to incorporation into the archaeological deposit and several retouchers bore traces of minor recent damage. The retouchers from Taforalt show no great differences in the choice of bone or size of bone through time. With the exception of an isolated, longer find from the Late MSA R7, relatively small, on average 7 centimetres long, lightweight fragments of shafts of bone from mainly medium-sized animals were consistently selected for retouching artefacts during the MSA Aterian, late MSA and in a potentially post-MSA context. The presence of bone retouchers in some of the layers in Sector 2 corresponds to the intermittent occupation of the cave during the MSA and post-MSA. Higher numbers of these finds in layers R7 and R9, in association with heavily worked side scrapers, suggest a focus on retouching activities in Sector 2 during the late MSA.

The newly recovered bone retouchers from Taforalt demonstrate that early modern humans in the Maghreb had already begun to perceive bone debris as a useful raw material for creating and modifying stone tools during the MSA Aterian, at around 85 ka. Interestingly, perforated *Nassarius* shells used as beads [28,43] were recovered from levels at the base of the Sector 2 deposits and date to approximately the same period as the oldest bone retouchers from the site. This shows that the use of bone retouchers at Taforalt formed part of a suite of MSA technologies—including the collection of shells to make beads by deliberate perforation or, perhaps less likely, selection for shells with a large natural perforation—that were established by 85 ka at least. Whereas evidence for perforated *Nassarius* shells disappears from Taforalt during the MSA Aterian at around 73 ka [28], bone retouchers at Taforalt continued to feature as an important tool, particularly into the late MSA, and are still apparent during the subsequent transitional period, which coincides broadly with the Late Glacial Maximum.

An intriguing aspect of the MSA lithic assemblages at Taforalt is the very low number of stone objects from Sector 2 also showing traces of use deriving from the shaping or refining of stone tools. In fact, only one hammerstone has been identified so far, in the Late MSA layer R7. Interestingly, the average length of hammerstones at some MSA sites is around 7cms. and more or less the same (7–8 cms.) at some sites with earlier Acheulian technologies in Morocco (pers. comm. 2019 Abdeljalil Bouzouggar). Considering this, the deliberate selection of numerous bone fragments similar in size to hammerstones for use as retouchers, may derive from a paucity of suitably-sized stones in the landscape around the cave. A Levallois flake from the Algerian site of Retaïme [44], which bears a few impact marks on the bulb -*“bulbe piqueté”*- is an interesting, but in North African contexts, rare example of a strategy where flint tools were utilised as retouchers [45], perhaps also reflecting a paucity of suitable materials at other sites.

There is no evidence at present for a continuation of the use of bone retouchers into the subsequent LSA phase of occupation at Taforalt. Rich assemblages of Iberomaurusian (LSA) finds have been recovered from Sectors 8 and 10 in the cave (Fig 1) [46,47,48] but none of the faunal remains recorded from these areas show unequivocal retoucher damage [49]. Recent and historic excavations of Iberomaurusian deposits at Taforalt have yielded a particularly rich assemblage of over 500 bone tools [50,51]. These comprised mainly pointed objects used for a variety of functions but, once again, no bone retouchers. Although a bone industry has been shown to be present at several Iberomaurusian sites, the published assemblages contain few tool forms and no bone retouchers [52,53]. Retouchers are, however, known from Iberomaurusian levels at Ifri N’Ammar, but significantly these are only made of stone [54]. Stone retouchers have not been identified so far from LSA deposits at Taforalt.

Although there is clear evidence at Taforalt of the use of bone retouchers over a long period of time from the MSA Aterian to the Late MSA, it is still uncertain whether the use of bone for these expedient tools, rather than stone, reflects an economic decision taken by MSA populations. So far, our knowledge of the presence and distribution of bone retouchers in North Africa is not extensive and the absence of bone retouchers at some sites may simply mean they have not been recognised. Currently, the finds from Taforalt represent one of the most clear and convincing assemblages of retouchers in the African records and are an important contribution to the non-lithic technologies of the MSA and the Aterian in particular.

## Supporting information

S1 File Description of bone retouchers from other parts of Africa. (DOCX)

S2 File Description of the lithic industries from Taforalt. (DOCX)

S3 File Description and analysis of the faunal assemblage from Sector 2 at Taforalt. (DOCX)

S4 File Terminology and conventions of the bone retouchers from Taforalt. (DOCX)

S1 Table Counts of identifiable faunal remains in the MSA and transitional deposits at Taforalt. Layers which produced only unidentifiable remains or no faunal remains at all are not included. LLG: finds from the Lower Laminated Group not attributable to a specific layer in this unit.
(TIF)

S2 Table Counts of faunal remains according to size-groups in the MSA and transitional deposits at Taforalt. Small size e.g. fox; medium size e.g. gazelle; medium-large e.g. Barbary sheep, alcelaphines, hyaena, bear; large size e.g. equids, large bovines, rhinoceros. Counts also include all identifiable faunal remains listed in Table A. LLG: finds from the Lower Laminated Group not attributable to a specific layer in this unit.
(TIF)

S3 Table Skeletal representation of faunal remains in the MSA and transitional deposits at Taforalt. Cr: cranium; man: mandible; pr: proximal; diaph: diaphysis; ds: distal; unid.: unidentifiable diaphyseal fragments of long bones; phal: phalanges.
(TIF)

S1 Fig Schematical diagram of bone retoucher TAF04-757 depicting orientation, nomenclature and descriptive criteria recorded on the Taforalt retouchers. (TIF)

S2 Fig Transitional, layer R3-4, (TAF04-757). Diagram of bone retoucher depicting observed features (a) photo of the find (b) scores and pits in use-area 1 superimposing cut marks and slight recent damage on right edge (c).
(TIF)

S3 Fig Late MSA, layer R7, (TAF05-2625). Snapshot of bone retoucher depicting observed features (a) photograph of the find (b) and details of use-area and cut marks (c).
(TIF)

S4 Fig Late MSA, layer 7 (TAF05-3243). Snapshot of bone retoucher depicting cut marks and flake scars on medial face (a) use-area, flake scar and cut marks on cranial face (b).
(TIF)

S5 Fig Late MSA, layer R9, (TAF04-1227). Snapshot of bone retoucher depicting observed features (a) photo of find (b) detail of intensive, deep scoring and pits in use-area 1 superimposing a cut mark (c).
(TIF)

S6 Fig Late MSA, layer R9, (TAF05-2491). Photo of bone retoucher depicting observed features (a) detail of use-area, and areas of modification located below the use-area (b).
(TIF)

S7 Fig Late MSA, layer R9, (TAF05-3328). Snapshot of bone retoucher depicting use-areas 1, 2 and 3 and other details.
(TIF)

S8 Fig MSA Aterian, layer 23, (TAF10-10172). Photographs of bone blank depicting use-area (a) and scoring (b).
(TIF)
