# Supplementary material for: Bone retouchers and technological continuity in the Middle Stone Age of North Africa
Source: PLoS One. 2020 Mar 30;15(3):e0230642. doi: 10.1371/journal.pone.0230642 (PMC7105130; doi:10.1371/journal.pone.0230642)
Supplement: S1 File — (DOCX) [file pone.0230642.s001.docx]

**Bone retouchers and technological continuity in the Middle Stone Age of North Africa**

Elaine Turner, Louise Humphrey, Abdeljalil Bouzouggar, Nick Barton

**S1 Description of Bone Retouchers from other parts of Africa**

The history of bone retouchers on the African continent is less well-established than on the European continent, even though the oldest evidence for bones interpreted as soft hammers from Olduvai Gorge, Tanzania, dates to between 2.1 -1.15 million years before present (Ma BP) **[1].** Early use of pressure flaking at around ~ 75 ka BP has been postulated for stone artefacts from Middle Stone Age (MSA) deposits at the South African site of Blombos Cave **[2],** and for serrated artefacts older than 70 ka BP at Umhlatuzana Rock Shelter [3,4] evidence of the types of tools required to carry out this technique is not abundant. Until recently, few MSA sites had yielded any unequivocal evidence of the complex bone technologies usually associated with the emergence of cognitive complexity [5], let alone bone retouchers. In the meantime, it has become accepted that formal bone tools were produced at South African and sub-Saharan sites during the MSA. The assemblage of worked bone from the cave site of Sibudu, for example, comprises 23 bone artefacts (pins, notched pieces, smoothers, pièces esquillées, awls, wedges and a possible projectile point) identified from Pre-Still Bay, Howieson’s Poort, Post-Howiesons’s Poort and Final MSA levels, representing the period between ~ 70 ka BP – 38 ka BP [6,7] Three broken bone flakes with naturally pointed ends – displayed wear patterns on the tips and may have been used as pressure flakers [7]. Recently, during analysis of an unpublished collection of bifacial serrated points from a sedimentary context preceding 77 ka BP at Sibudu Cave [8] a fragment of a bone compressor and a bone percussion tool were recovered in a single layer (layer Caspar). The latter tool bears scores and scrapes on the surface of the bone comparable to the traces identified in this paper as retoucher damage; a quartzite flake fragment is still embedded in one of the scores [8, Fig 14].

At Blombos Cave, an assemblage of 28 bone tools from MSA levels, dating to around 70 ka, was analysed, but only one find, from the BBC M1/2 phase, bore distinctive traces of retoucher use on the shaft [9, Appendices A1 and A2, SAM-AA No. 8950, “percussor”]. This find [9, Fig 10a – c], is a large shaft fragment from the metacarpal of a size IV bovid, probably eland. Two areas of use are visible on the surface of the find, located towards the apex and base of the bone respectively. The upper area is characterised by multiple, elongated scores, several deep pit-like features and scaling (loss of patches of the surface of the bone) [10] pointing to a fairly intensive use of this part of the bone as a retoucher

Nine additional bone artefacts from Blombos from the M1 phase (Still Bay Complex), dated by Optically Stimulated Luminescence (OSL) and Thermoluminescence (TL) to about 75 – 77 ka (OIS 5a) were analysed subsequently [5]. These finds included a well-preserved, midshaft fragment of a long bone from an unknown bovid [5, Fig 5] which has closely spaced, linear scores towards one end of the bone, indicative of its occasional use as a retoucher. This particular find may have been used for different activities. Microscopic analysis of contiguous, possibly anthropogenic, scaled removals along the upper edge of the find, similar to those found on an end-scraper, revealed light smoothing that can be attributed to use-wear. In addition, its surface bore a set of six, mainly oblique, incisions produced by a sharp lithic point. One of these incisions overlaid the linear scores, indicating that after a short period of use as a retoucher, the bone appears to have been deliberately incised [5].

For North Africa, in particular the Maghreb, the picture seems to be more diffuse, with very low counts in MSA levels of bone artefacts in general and expedient tools, such as bone retouchers, in particular. Two objects, one made from elephant or hippopotamus ivory and shaped as a point, the other a small ivory plaquette [11] from the lowest Aterian assemblage (Level I) at Dar es-Soltan 1, indicate an early appearance of worked organic remains probably earlier than 110,000 years ago, during the later stages of MIS 5 [12] More recently, a bone knife was recovered from a layer that can be securely dated to ~90 ka during excavations at Dar es-Soltan 1 [13] El Hajraoui (14) described four worked bones from levels 5 and 6 (Unit 8) at the cave site of El Mnasra, which belong to the MSA Aterian. Traces of scraping and abrasion were observed on three elongated bones and on a smaller bone flake [14, Fig 3]. Two of the finds show linear striations on their surfaces, which may have been produced during use [14, Fig 3a] or as the remains of organic tissues were cleaned from the bone prior to use [14, Fig 3b], but none of these finds bore typical retoucher damage. During a re-analysis of faunal remains from El Mnasra, Campmas [15, Figs 217-234] described seven pieces of worked bone, but again none of these finds displayed distinct areas of use-wear on their surfaces, comparable to that found on bone retouchers, although two were considered as bone knives [13]. The finds from El Mnasra were also published in a work by El Hajraoui and Debénath [16].

In contrast, the bone industry from a second site analysed by Campmas, El Harhoura 2, comprised three bone retouchers [15: Fig 147, Table 123], one each from the MSA layers 4A, 5 and 8. The retouchers are all on long bone diaphyses; the find from layer 5 could be identified more closely as a metatarsus of an alcelaphine. Unfortunately, the dating of the MSA at El Harhoura 2 and El Mnasra is problematic. Although both sites have long sedimentary sequences extending back to MIS 5, and are partly contemporaneous, the results of dating from the sites differ significantly, depending on the method applied (OSL and Electron-Spin-Resonance [ESR]- Uranium Series [US]) [16]. So far, the combined age for the MSA layers at both sites lies between 133.2 ± 7 ka and 44.0 ± 3 ka [17]. A small number of bone retouchers has also been identified from MSA layers at Contrebandiers (pers. comm. 2018, Emily Hallet-Desguez).

In summary, the presence of bone retouchers at African sites is meagre. So far, there is a single example published recently from Sibudu Cave older than 77 ka, two bone retouchers published from the south African site of Blombos, dating to between 77 – 75 ka, three bone retouchers from the Moroccan site of El Harhoura 2, dating to between ~123 - 44 ka and a small number of unpublished finds from the site of Contrebandiers.

References

1. Backwell LR, d’Errico F. The first use of bone tools: a reappraisal of the evidence from Olduvai Gorge, Tanzania. Palaeontol. Africa 2005; 40: 95-158.

2. Mourre V, Villa P, Henshilwood CS. Early use of pressure flaking on lithic artifacts at Blombos cave, South Africa. Science 2010; 330: 659 – 662.

3. Högberg A, Lombard M. Indications of Pressure Flaking more than 70 thousand years ago at Umhlatuzana Rock Shelter. South African Archaeological Bulletin 2016; 71: 53–59.

4. Lombard M, Wadley L, Jacobs Z, Mohapi M, Roberts RG. Still Bay and serrated points from Umhlatuzana Rock Shelter, KwaZulu-Natal, South Africa. J Archaeol Sci. 2010; 37: 1773–1784.

5. d’Errico F, Henshilwood CS. Additional evidence for bone technology in the southern African Middle Stone Age. J Hum Evol. 2007; 52: 142–163.

6. Backwell L, d’Errico F, Wadley L. Middle Stone Age bone tools from the Howieson’s Poort layers, Sibudu Cave, South Africa. J Archaeol Sci. 2008; 35: 1566–1580.

7. d’Errico F, Backwell, LR, Wadley L. Identifying regional variability in Middle Stone Age bone technology: The case of Sibudu Cave. J Archaeol Sci. 2012; 39, 1–17.

8. Rots V, Lentfer C, Schmid VC, Porraz G, Conard NJ. Pressure flaking to serrate bifacial points for the hunt during the MIS5 at Sibudu Cave (South Africa). PLoS One 2017; 12(4). doi:10.1371/journal.pone.0175151.

9. Henshilwood CS, d’Errico F, Marean CW, Milo RG, Yates R. An early bone tool industry from the Middle Stone Age at Blombos Cave, South Africa: implications for the origins of modern human behaviour, symbolism and language. J Hum Evol. 2001; 41: 631–678

10. Mallye J-B, Thiébaut C, Mourre V, Costamagno S, Claud É, Weisbecker P. The Mousterian bone retouchers of Noisetier cave: experimentation and identification of marks. J Archaeol Sci. 2012; 3: 1131 – 1142.

11. Ruhlmann A. La grotte préhistorique de Dar es-Soltan. Collection Hésperis 11. Institut des Hautes Études Marocaines 1951: 1–210.

12. Barton RNE, Bouzouggar A, Collcutt SN, Schwenninger JL, Clark-Balzan L. OSL dating of the Aterian levels at Dar es-Soltan I (Rabat, Morocco) and implications for the dispersal of modern Homo sapiens. Quaternary Science Reviews 2009; 28: 1914–1931.

13. Bouzouggar A, Humphrey LT, Barton N, Parfitt SA, Clark Balzan L, Schwenninger J-L, et al. 90,000 year-old specialised bone technology in the Aterian Middle Stone Age of North Africa. PLOS 2018[, doi.org/10.1371/journal.pone.0202021](file:///C:\Users\rneba\AppData\Local\Microsoft\Windows\INetCache\Content.Outlook\CAA1VYOQ\,%20doi.org\10.1371\journal.pone.0202021).

14. El Hajraoui MA. L’Industrie osseuse Atérienne de la Grotte d’El Mnasra (Région de Temara, Maroc). Préhistoire Anthropologie Méditerranéennes.1994; T.3: 91 – 94.

15. Campmas E. Caractérisation de l’occupation des sites de la région de Témara (Maroc) au Pléistocène supérieur et nouvelles données sur la subsistance des Hommes du Paléolithique moyen d’Afrique du Nord : Exemples des approches taphonomiques et archéozoologiques menées sur les faunes d’El Harhoura 2 et d’El Mnasra. Ph. D. Thesis, The University of Bordeaux. 2012.

16. El Hajraoui ME, Debénath A, 2012. L’Industrie osseuse. In: El Hajraoui MA, Nespoulet, R, Debénath A, Dibble HL. (Eds.), Préhistoire de la Région de Rabat-Temara. Villes et sites archéologiques du Maroc (V.E.S.A.M.) 2012. Ministère de la Culture, Institut National des Sciences de l’Archéologie et du Patrimoine, Rabat; Volume III: 179-188.

17. Stoetzel E, Campmas E, Michel P, Bougariane B, Ouchaou B, Amani F, El Hajraoui ME, Nespoulet R. Context of modern human occupations in North Africa: Contribution of the Témara caves data. Quaternary International 2014; 320: 143 – 161.
