# Supplementary material for: Bone retouchers and technological continuity in the Middle Stone Age of North Africa
Source: PLoS One. 2020 Mar 30;15(3):e0230642. doi: 10.1371/journal.pone.0230642 (PMC7105130; doi:10.1371/journal.pone.0230642)
Supplement: S2 File — (DOCX) [file pone.0230642.s002.docx]

**Bone retouchers and technological continuity in the Middle Stone Age of North Africa**

Elaine Turner, Louise Humphrey, Abdeljalil Bouzouggar, Nick Barton

**S2 Description of the lithic industries**

Bone retouchers have been recovered from layers R3-4, R7, R9, R10, R16 and R23c and e and lithic artefacts occur in each of these layers. The majority of the layers contain evidence of MSA industries typical of those found in North Africa with common use of Levallois technology [1, 2]. The exception is the assemblage from layers R3 – R4 which is made up of flakes including thinning flakes and large, parallel-sided bifacial forms [3] that we refer to as bifacial adzes [3]. There is no clear evidence for the use of the Levallois technique, though MSA-type side scrapers have been recorded from equivalent layers elsewhere in the cave [4]. Amongst the notable finds from layers R7 and R9 are representative examples of side scrapers (20) which are the most common tool forms. The scrapers are associated with Levallois flakes often with faceted butts, and included in the debitage are preferential radial flake cores. In contrast Layer R10 is impoverished in lithic artefacts. Of the very few examples are Levallois flakes but there are no cores or retouched tools. The sequence continues downwards through the Pink Group and the underlying Lower Laminated Group which includes layer R16. This layer has a very rich lithic assemblage. It comprises a high Levallois component with flake cores (preferential radial) as well as discoidal and multiplatform core types. The debitage also contains a range of Levallois products such as flakes, laminar flakes and bladelets. Apart from a variety of single and double sidescrapers (11), the tool assemblage includes a pedunculate point, a unifacial foliate and four other bifacial and unifacial tools typical of the Aterian facies. Further assemblages have been recovered in R23. The two bone retouchers from this layer come from sub-units 23c and 23e. Layer 23c is not particularly rich in lithic artefacts but contains examples of Levallois flakes. Layer 23e has few cores, but many Levallois flakes, laminar flakes and bladelets. Side scrapers (3) are the main recognisable retouched tool from this sub-unit.

**References**

1. Bouzouggar A, Barton RNE, 2012. The identity and timing of the Aterian in Morocco. In: Hublin J-J, McPherron S. editors. Modern origins: A North African perspective*.* Dordrecht: Springer. pp. 93-105.

2. Dibble HL, Aldeias V, Jacobs Z, Olszewski DI, Rezek Z, et al. On the industrial attributions of the Aterian and Mousterian of the Maghreb. J Hum Evol. 2013; 64: 194-210.

3. Barton RNE, Bouzouggar A, Hogue JT, Lee S, Collcutt SN, et al. Origins of the Iberomaurusian in NWAfrica: New AMS radiocarbon dating of the Middle and Later Stone Age deposits at Taforalt Cave, Morocco. J Hum Evol. 2013, [65(3):](http://www.sciencedirect.com/science/journal/00472484/65/3) 266–281.

**4. Clark, J.D. Kalambo Falls Prehistoric Site. The Earlier and Middle Stone Age. Cambridge. Cambridge University Press. p. 39.**
