# Supplementary material for: Bone retouchers and technological continuity in the Middle Stone Age of North Africa
Source: PLoS One. 2020 Mar 30;15(3):e0230642. doi: 10.1371/journal.pone.0230642 (PMC7105130; doi:10.1371/journal.pone.0230642)
Supplement: S3 File — (DOCX) [file pone.0230642.s003.docx]

**Bone retouchers and technological continuity in the Middle Stone Age of North Africa**

Elaine Turner, Louise Humphrey, Abdeljalil Bouzouggar, Nick Barton

**S3 Description and analysis of the faunal assemblage from Sector 2**

The industries are associated with well-preserved, but highly fragmentary faunal remains, which show clear evidence of human interaction in the form of butchery marks (cut marks; marks of impact produced when the bones were deliberately opened to retrieve marrow).

Out of a total of 752 faunal remains recorded in Sector 2 (including finds from layers R3 – R23 and finds from the Lower Laminated Group not attributable to a specific layer within this set), only 101 could be identified (Table S3 A). Barbary sheep (*Ammotragus lervia*) is the dominant species and identifiable remains of this animal are present in most of the layers in S2. The dominance of Barbary sheep reflects the location of the cave in the Beni-Snassen hills, surrounded by stony plateaus, steep valley slopes and coarse wadi bottoms, habitats favoured by this species [1]. Remains of a large bovine, equids, gazelle and members of the alcelaphines, which in some cases could be identified to hartebeest, are also present. Less common are rhinoceros, hyaena, bear and fox in the MSA Aterian levels and a small carnivore (possibly fox) in the late MSA levels. The overlying layers R3 - R4 with an undiagnostic lithic industry have produced only the remains of equids. Since the proportion of fragmentary bones at Taforalt was high, size groups were also utilised to quantify the material so that finds not identifiable to taxon could also be included. Bones were attributed to size group using overall size of the find (length x breadth) and cortical bone thickness. The highest counts are from the medium-large size category (Table S3 B). Although different animals are included in this group, the high representation of remains of Barbary sheep among the identifiable finds makes it likely that the bulk of the fragments in the medium-large size group also belong to this species.

This pattern of faunal representation, dominated by Barbary sheep with lower and fluctuating counts of large bovines, equids, gazelle and alcelaphines, has been observed in both the MSA and LSA levels in the cave, suggesting that procurement of game changed very little over time at Taforalt [2]. The skeletal representation of the faunal remains (Table C) shows large counts of diaphyseal fragments from limb bones, foot bones and not further identifiable long bones.

During analysis, an extensive range of criteria was entered into a data-base. These data ranged from basic excavation information and the dimensions of the find, to species and bone identification and details of modifications of the finds. The surfaces of all of the bones from Taforalt, including the retouchers were examined for traces of modification using a hand-held magnifying lens (8x). The surfaces of the bones from Sector 2 were exceptionally well-preserved, surficial marks were easily observed and the low magnification of the lens was deemed sufficient to verify the identification of the marks on the find. Identification criteria summarised by Fisher [3] were used to determine modifications produced by humans, such as cut marks and scrapes produced by stone tools, along with conchoidal flake scars created by cracking open bones with hard objects in order to extract marrow, and marks produced by nonhuman processes, such as carnivore tooth scoring. The orientation of cut marks or scrapes – transverse, oblique or longitudinal - was recorded relative to the long axis of the find. Binford’s [4] chapter on the form and placement of cut marks on specific bones was primarily used to attribute cut marks on the bones to a particular butchering activity (e.g. disarticulation or filleting). Traces of burning were also observed and described using stages defined by Stiner et al. [5]. The fracture edges of the bones were classified as green (fresh) or dry, using criteria given by Johnson [6]. Post-depositional modifications, such as pitting of the surface, which may be an initial stage of cave corrosion [7: 101] were also recorded, as well as recent damage; the latter easily identified by a very distinctive change of colour: intact bones from S2 are brown, areas of recent damage or recent fractures are pale brown to cream in colour.

**References**

1. Kingdon J. The Kingdon Field Guide to African Mammals. Natural World. San Diego, London, Boston, New York, Sydney, Tokyo, Toronto: Academic Press; 1997.

2. Turner E. Large Mammalian Fauna. In: Barton RNE, Bouzouggar A, Collcutt S, Humphrey L, editors. Cemeteries and Sedentism in the Later Stone Age of NW Africa: Excavations at Grotte des Pigeons, Taforalt. Monographien des RGZM, 2019, 147 Schnell & Steiner, pp. 239 -308.

3. Fisher JW Jr. Bone surface modifications in archaeology. Journal of Archaeological Method and Theory 1995; Vol. 2, No. 1: 7 – 68.

4. Binford LR. Bones. Ancient men and modern myths. New York: Academic Press; 1981.

5. Stiner MC, Kuhn SL, Weiner S, Bar-Yosef O. Differential burning, recrystallization, and fragmentation of archaeological bone. J Archaeol Sci. 1995; 22 (2): 223 - 237.

6. Johnson E. Current developments in bone technology. In: Schiffer MB. (Ed.), Advances in Archaeological Method and Theory 1985; 8: 157 – 234.

7. Andrews P. Owls, Caves and Fossils. London: The Natural History Museum; 1990.

**S3 Table A. Counts of identifiable faunal remains in the MSA and Transitional deposits at Taforalt.** Layers which produced only unidentifiable remains or no faunal remains at all are not included. LLG: finds from the Lower Laminated Group not attributable to a specific layer in this unit.

**S3 Table B. Counts of faunal remains according to size-groups in the MSA and Transitional deposits at Taforalt.** Small size e.g. fox; medium size e.g. gazelle; medium-large e.g. Barbary sheep, alcelaphines, hyaena, bear; large size e.g. equids, large bovines, rhinoceros. Counts also include all identifiable faunal remains listed in Table A. LLG finds from the Lower Laminated Group not attributable to a specific layer in this unit.

**S3 Table C. Skeletal representation of faunal remains in the MSA and Transitional deposits at Taforalt.** Cr: cranium; man: mandible; pr: proximal; diaph: diaphysis; ds: distal; unid.: unidentifiable diaphyseal fragments of long bones; phal: phalanges.
