# Supplementary material for: Bone retouchers and technological continuity in the Middle Stone Age of North Africa
Source: PLoS One. 2020 Mar 30;15(3):e0230642. doi: 10.1371/journal.pone.0230642 (PMC7105130; doi:10.1371/journal.pone.0230642)
Supplement: S4 File — (DOCX) [file pone.0230642.s004.docx]

**Bone retouchers and technological continuity in the Middle Stone Age of North Africa**

Elaine Turner, Louise Humphrey, Abdeljalil Bouzouggar, Nick Barton

S4 Analysing and recording the bone retouchers from Taforalt

In the main, the bone retouchers were recorded and described using conventions and terminology proposed by Taute [1] and Mallye et al. [2], with minor adjustments to accommodate criteria observed on the finds from Taforalt. The greatest length of the find was taken as the long axis of the bone. Since the bulk of the retouchers from Taforalt comprise the shafts of long bones, the greatest length corresponds to the orientation of the element in the skeleton. The terms top, mid, base, left lateral and right lateral were used for purposes of orientation when describing the location of features on the finds (S4 Fig A, left). The retoucher was oriented with the use-area placed at or towards the top. On finds where more than one use-area was present, the use-areas were numbered consecutively from the top, but in contrast to Mallye et al. [2, 1133], were not reoriented for the analysis of each individual area. Terms relating to the use areas and their traces were taken from Mallye et al. [2].

Details of all modifications observed on the Taforalt retouchers (e.g. cut marks, flake removals, use-areas and their traces) (S4 Fig A, right) were either drawn schematically onto photoshots of those finds which had been scanned (S4 Fig D), depicted by detailed photographs (S4 Fig H), or illustrated using a combination of these techniques (S4 Fig E), thus providing not only an essential framework for the description of the finds, but also for the recognition of the different stages of utilisation of the bone.

Figs S4 B-H illustrate selected retouchers from the MSA Aterian, Late MSA and transitional levels at Taforalt and details of their individual characteristics.

**References**

1. Taute W, Retouchere aus Knochen, Zahnbein und Stein vom Mittelpaläolithikum bis zum Neolithikum. Fundberichte aus Schwaben 1965; 17: 76 – 102.

2. Mallye J-B, Thiébaut C, Mourre V, Costamagno S, Claud É, Weisbecker P, The Mousterian bone retouchers of Noisetier cave: experimentation and identification of marks. J Archaeol Sci. 2012; 3: 1131 – 1142.

**S4 Fig. A.** Schematical diagram of bone retoucher TAF04-757 depicting orientation, nomenclature and descriptive criteria recorded on the Taforalt retouchers.

**S4 Fig B.** Transitional, layer R3-4, (TAF04-757). Diagram of bone retoucher depicting observed features (a) photo of the find (b) scores and pits in use-area 1 superimposing cut marks and slight recent damage on right edge (c).

**S4 Fig C.** Late MSA, layer R7, (TAF05-2625). Snapshot of bone retoucher depicting observed features (a) photograph of the find (b) and details of use-area and cut marks (c).

**S4 Fig D.** Late MSA, layer 7 (TAF05-3243). Snapshot of bone retoucher depicting cut marks and flake scars on medial face (a) use-area, flake scar and cut marks on cranial face (b).

**S4 Fig E.** Late MSA, layer R9, (TAF04-1227). Snapshot of bone retoucher depicting observed features (a) photo of find (b) detail of intensive, deep scoring and pits in use-area 1 superimposing a cut mark (c).

**S4 Fig F.** Late MSA, layer R9, (TAF05-2491). Photo of bone retoucher depicting observed features (a) detail of use-area, and areas of modification located below the use-area (b).

**S4 Fig G.** Late MSA, layer R9, (TAF05-3328). Snapshot of bone retoucher depicting use-areas 1, 2 and 3 and other details.

**S4 Fig H.** MSA Aterian, layer 23, (TAF10-10172). Photographs of bone blank depicting use-area (a) and scoring (b).
