# Supplementary figures and images for: Bone retouchers and technological continuity in the Middle Stone Age of North Africa
Source: PLoS One. 2020 Mar 30;15(3):e0230642. doi: 10.1371/journal.pone.0230642 (PMC7105130; doi:10.1371/journal.pone.0230642)

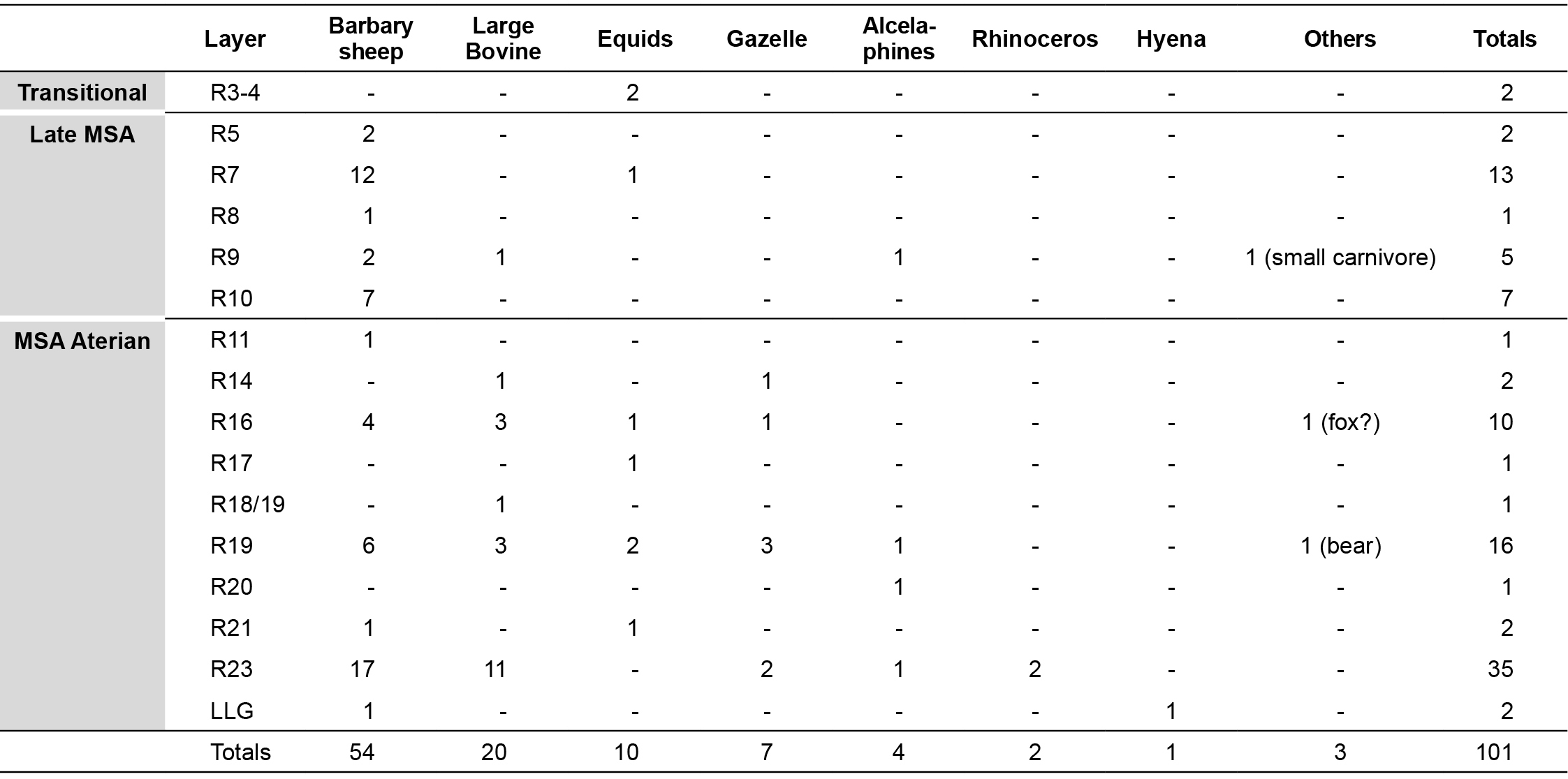

Supplement: S1 Table — Layers which produced only unidentifiable remains or no faunal remains at all are not included. LLG: finds from the Lower Laminated Group not attributable to a specific layer in this unit. (TIF) [file pone.0230642.s005.tif]

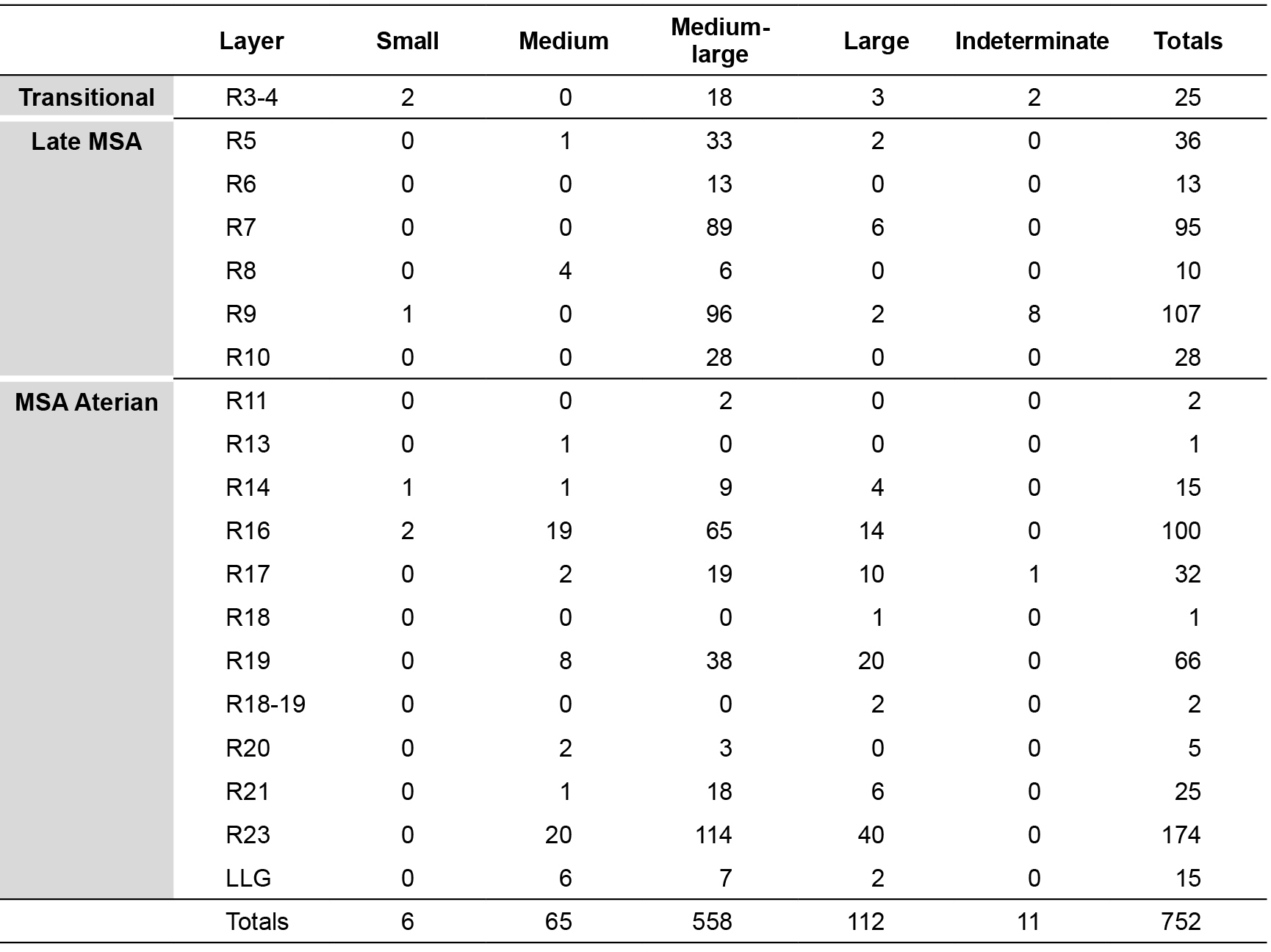

Supplement: S2 Table — Small size e.g. fox; medium size e.g. gazelle; medium-large e.g. Barbary sheep, alcelaphines, hyaena, bear; large size e.g. equids, large bovines, rhinoceros. Counts also include all identifiable faunal remains listed in Table A. LLG: finds from the Lower Laminated Group not attributable to a specific layer in this unit. (TIF) [file pone.0230642.s006.tif]

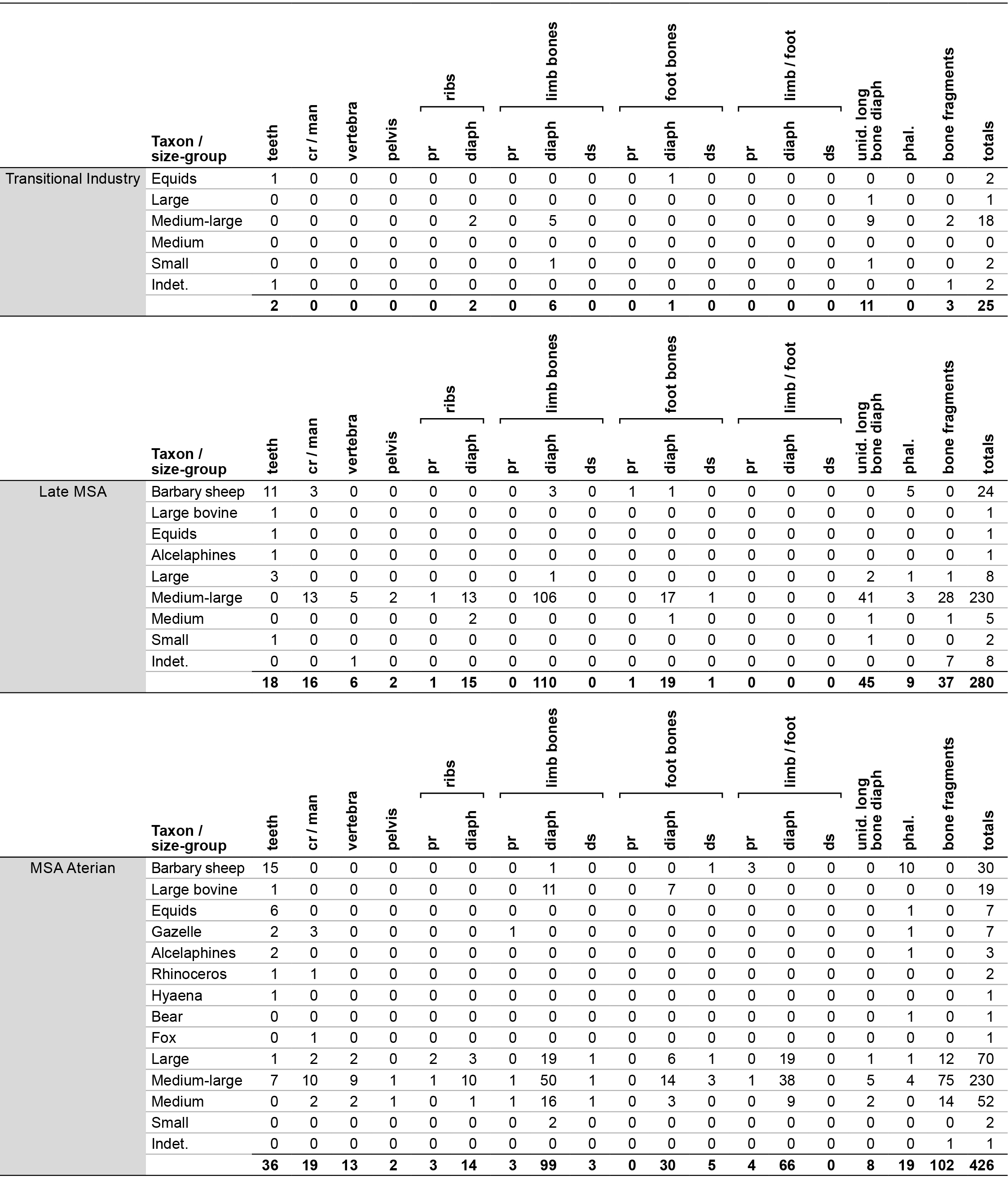

Supplement: S3 Table — Cr: cranium; man: mandible; pr: proximal; diaph: diaphysis; ds: distal; unid.: unidentifiable diaphyseal fragments of long bones; phal: phalanges. (TIF) [file pone.0230642.s007.tif]

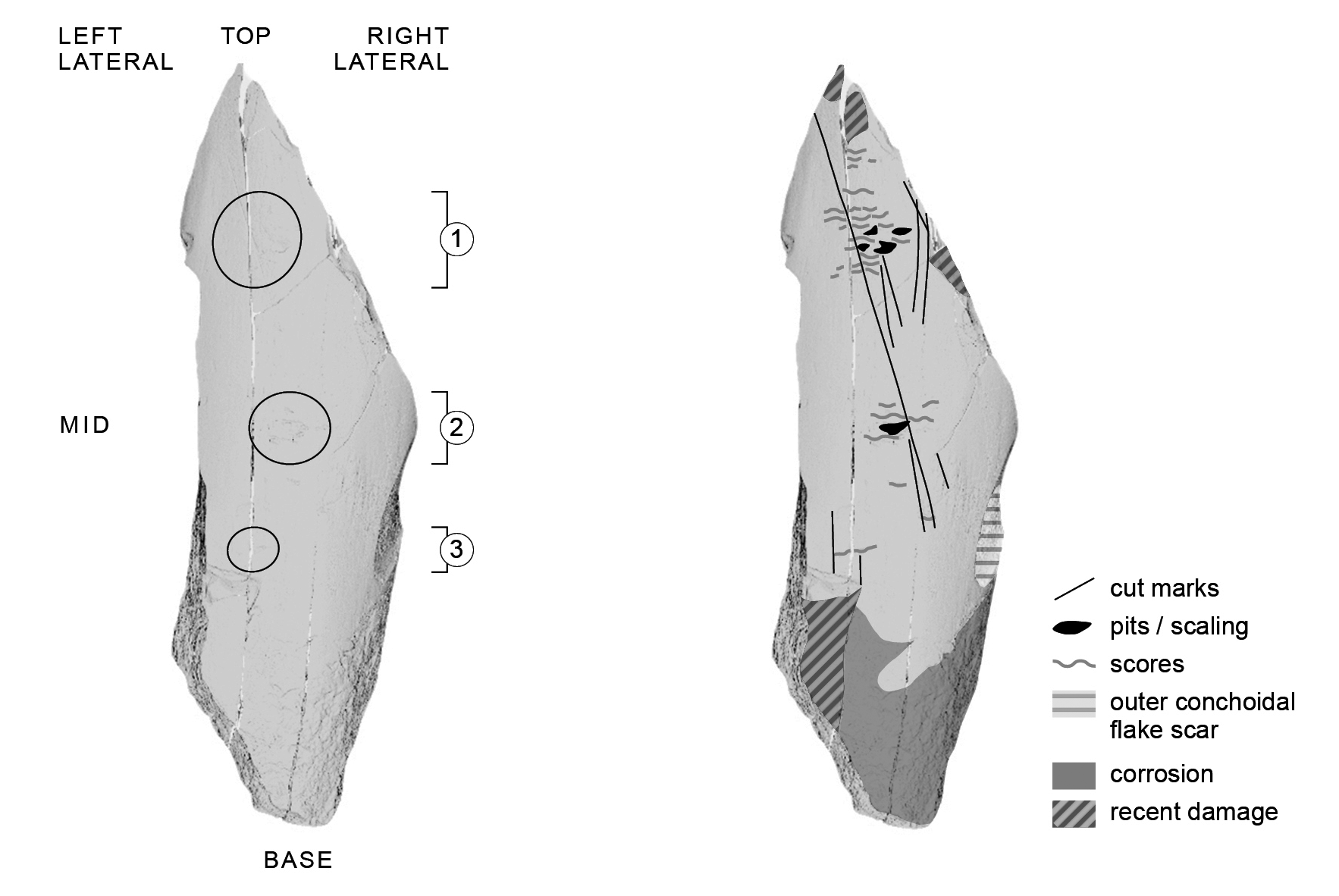

Supplement: S1 Fig — (TIF) [file pone.0230642.s008.tif]

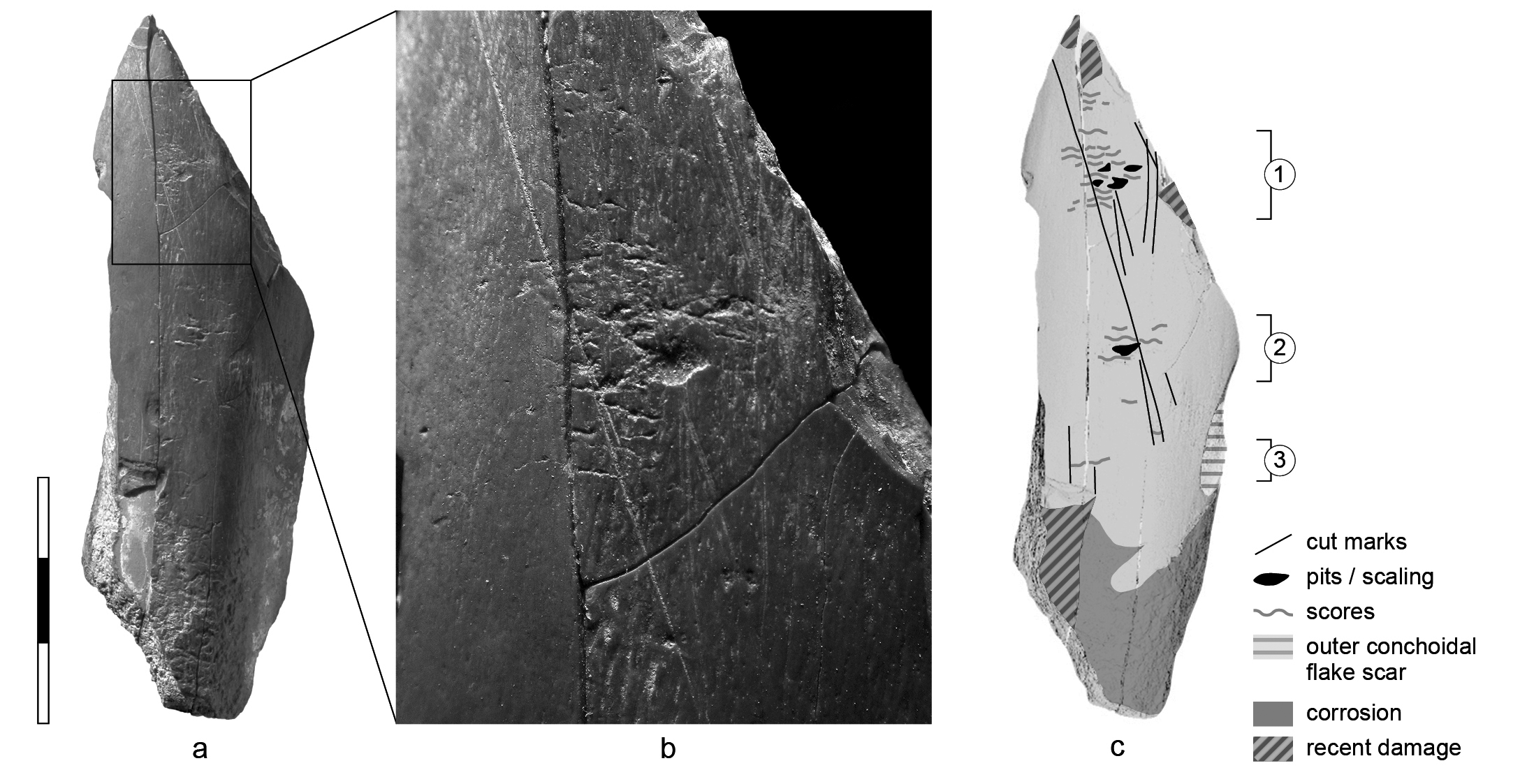

Supplement: S2 Fig — Diagram of bone retoucher depicting observed features (a) photo of the find (b) scores and pits in use-area 1 superimposing cut marks and slight recent damage on right edge (c). (TIF) [file pone.0230642.s009.tif]

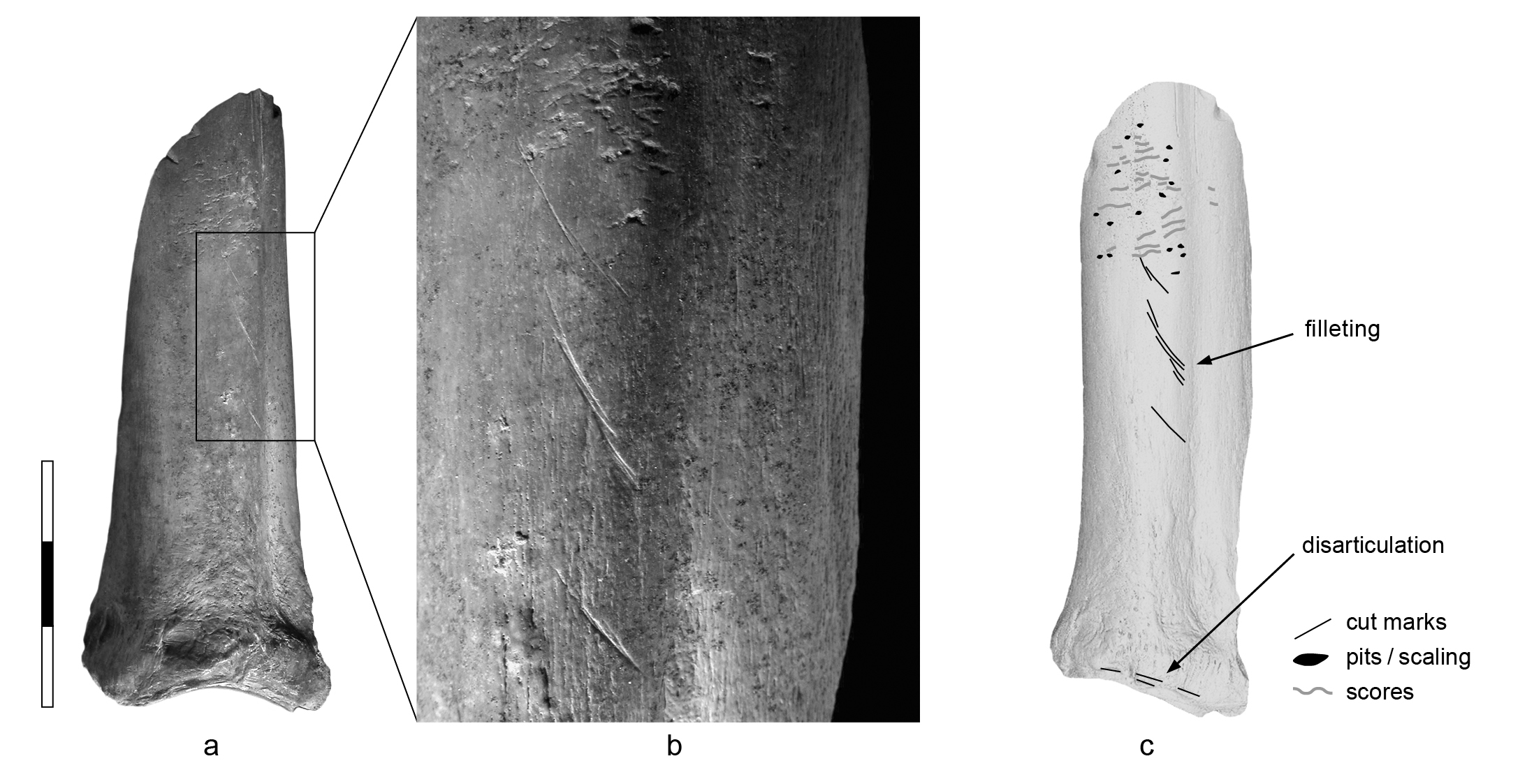

Supplement: S3 Fig — Snapshot of bone retoucher depicting observed features (a) photograph of the find (b) and details of use-area and cut marks (c). (TIF) [file pone.0230642.s010.tif]

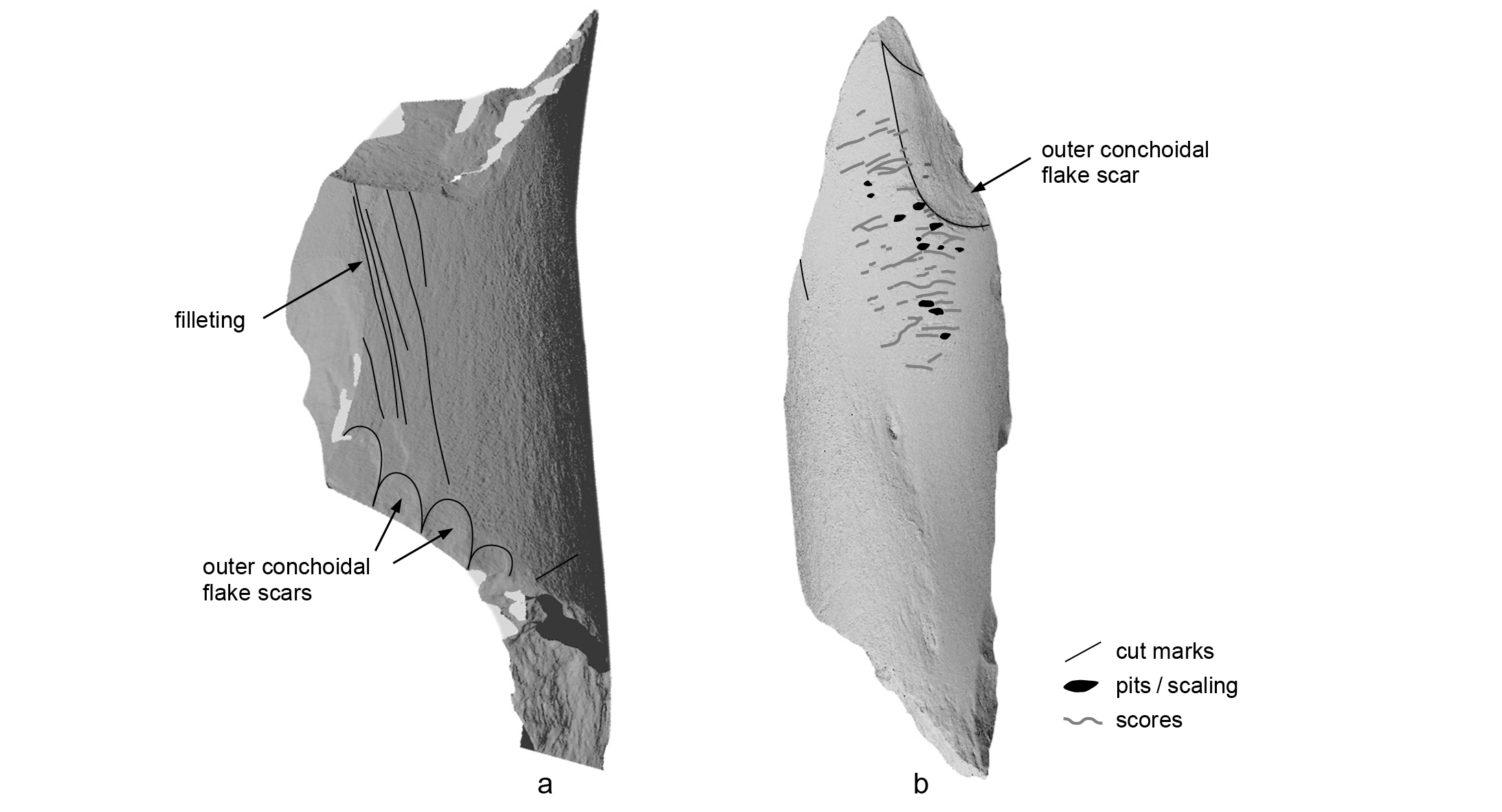

Supplement: S4 Fig — Snapshot of bone retoucher depicting cut marks and flake scars on medial face (a) use-area, flake scar and cut marks on cranial face (b). (TIF) [file pone.0230642.s011.tif]

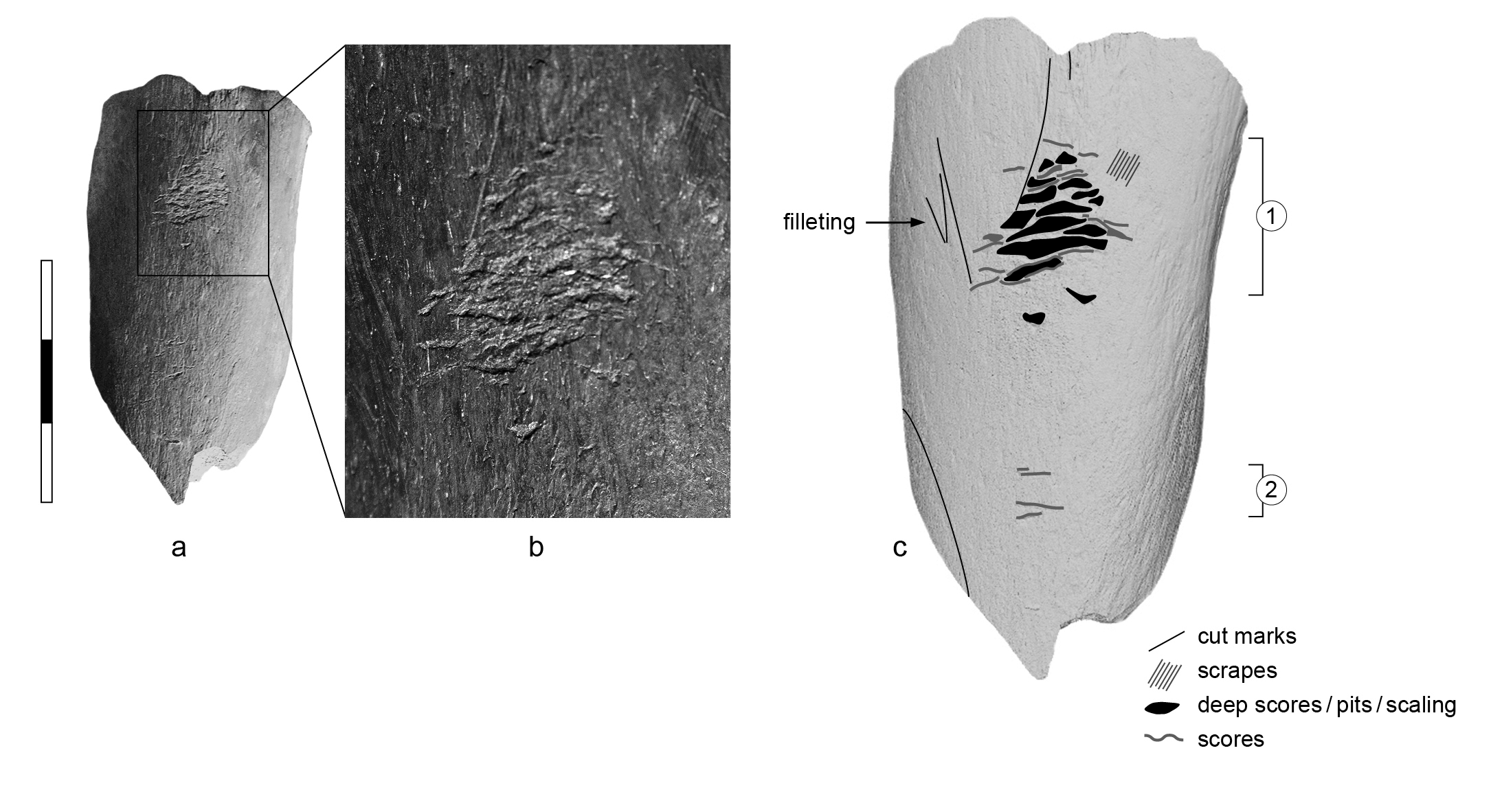

Supplement: S5 Fig — Snapshot of bone retoucher depicting observed features (a) photo of find (b) detail of intensive, deep scoring and pits in use-area 1 superimposing a cut mark (c). (TIF) [file pone.0230642.s012.tif]

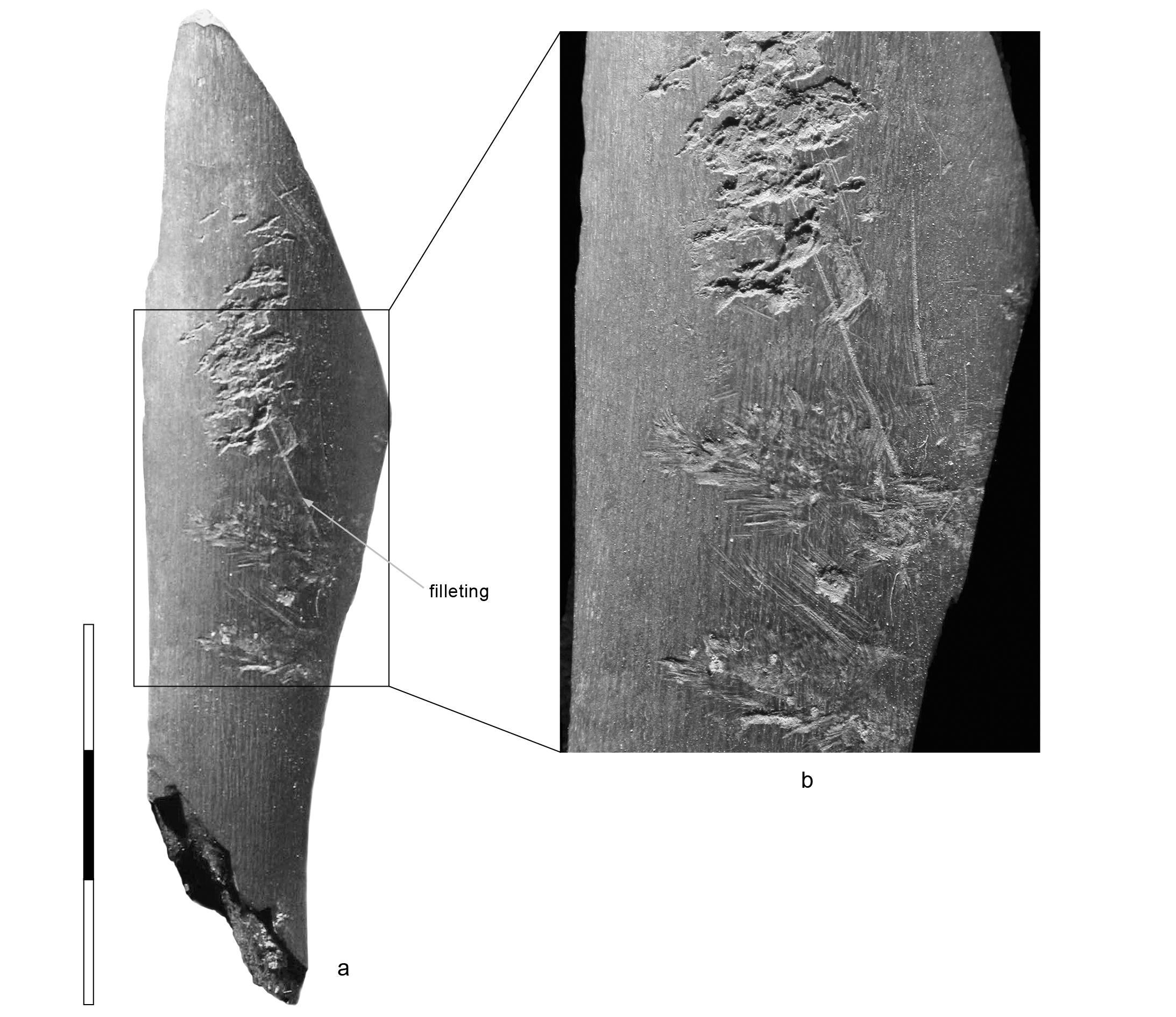

Supplement: S6 Fig — Photo of bone retoucher depicting observed features (a) detail of use-area, and areas of modification located below the use-area (b). (TIF) [file pone.0230642.s013.tif]

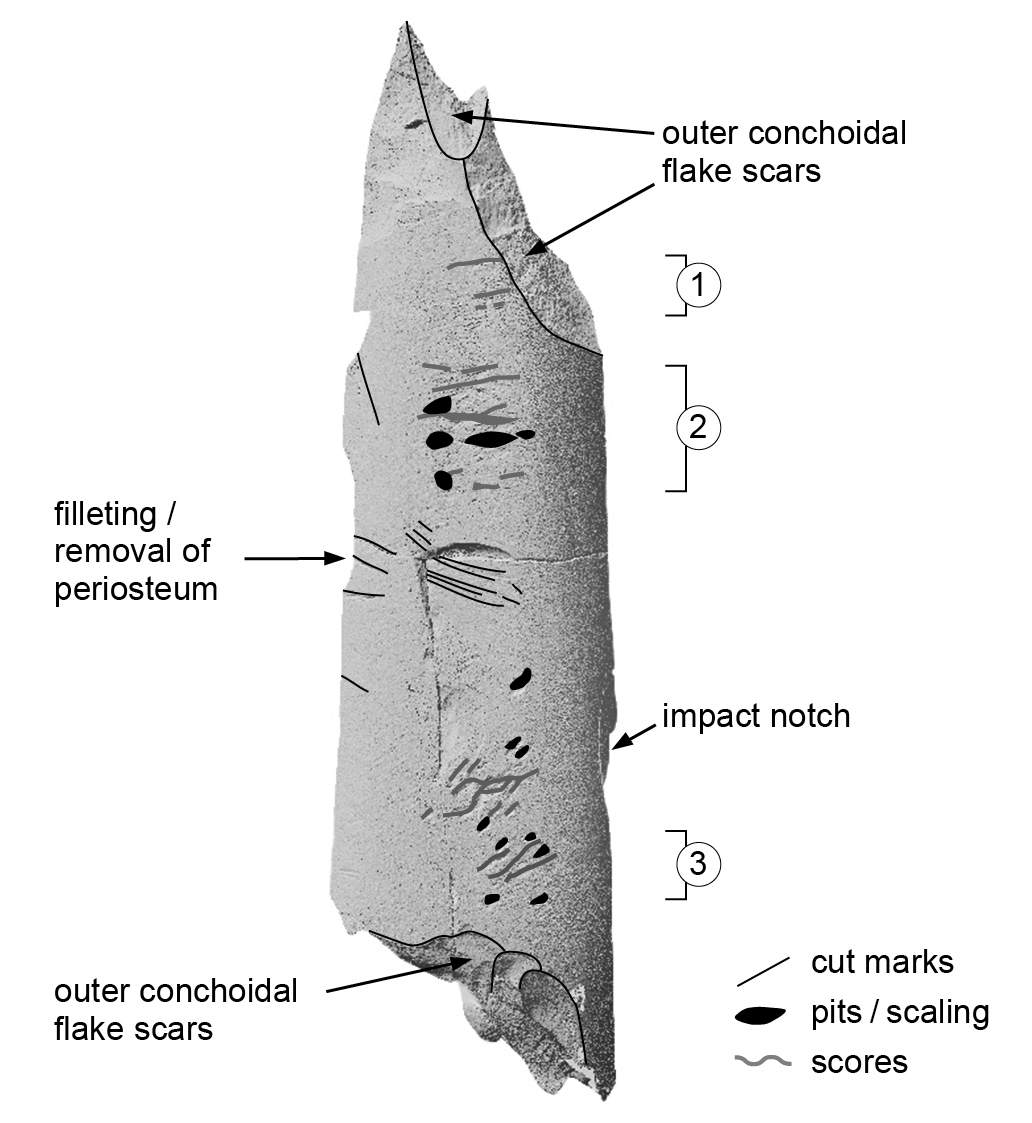

Supplement: S7 Fig — Snapshot of bone retoucher depicting use-areas 1, 2 and 3 and other details. (TIF) [file pone.0230642.s014.tif]

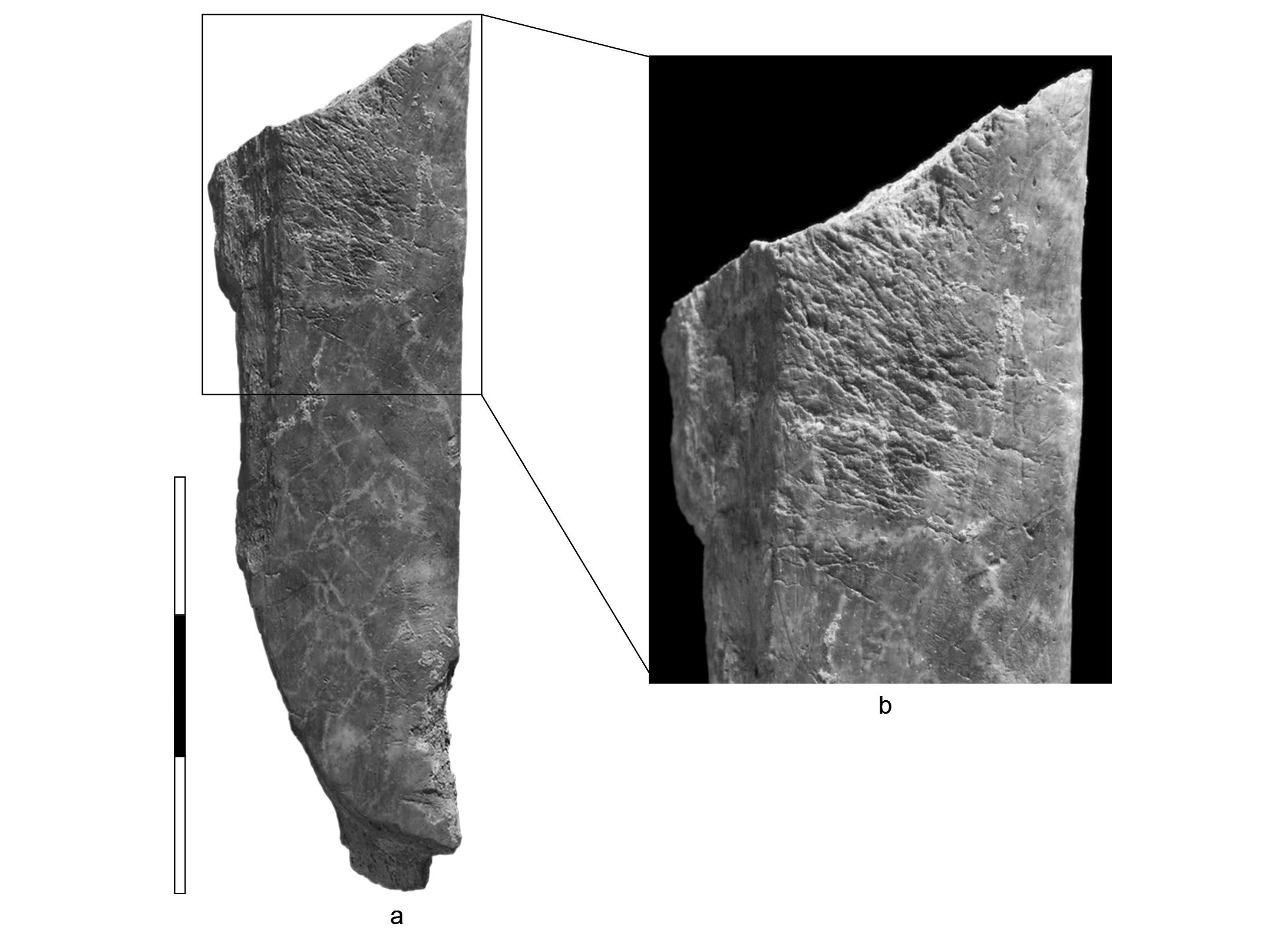

Supplement: S8 Fig — Photographs of bone blank depicting use-area (a) and scoring (b). (TIF) [file pone.0230642.s015.tif]
